# Targeting the NAT1‐ENO1‐Lactate Axis Destabilizes PD‐L1 to Reinvigorate Antitumor Immunity in Colorectal Cancer

**DOI:** 10.1002/mco2.70867

**Published:** 2026-07-16

**Authors:** Yi‐Xuan Liu, Wen‐Xin Wu, Yang Zang, Guang Hu, Xin Guo, Yi Wang, Yun Yang, Zhi Jiang, Xiu‐Ming Li, Feng Liu, Yue‐Yue Wu, Zu‐Da Pan, Yuan‐Meng Hu, Ruo‐Yang Zhang, Hai‐Yi Zhang, Ping‐An Chang, Xiao‐Shun He, Wen‐Juan Gan, Hua Wu

**Affiliations:** ^1^ Department of Pathology The Fourth Affiliated Hospital of Soochow University Suzhou Medical College of Soochow University Soochow University Suzhou Jiangsu China; ^2^ Medical College, Yangzhou University Yangzhou Jiangsu China; ^3^ Department of Bioinformatics School of Life Science Soochow University Suzhou Jiangsu China; ^4^ Biomedical Basic Research Center (BBRC) of Jiangsu Soochow University Suzhou Jiangsu China; ^5^ National Center of Technology Innovation for Biopharmaceuticals Suzhou Biomedical Industry Innovation Center Suzhou Jiangsu China; ^6^ Department of Biochemistry and Molecular Biology Suzhou Medical College of Soochow University Soochow University Suzhou Jiangsu China; ^7^ Department of Pathology The First Affiliated Hospital of Soochow University Suzhou Jiangsu China; ^8^ Department of General Surgery Suzhou Guangci Cancer Hospital Suzhou Jiangsu China; ^9^ Department of Urology Surgery Affiliated Dongtai Hospital of Nantong University Dongtai Jiangsu China; ^10^ Cancer Institute Suzhou Medical College of Soochow University Soochow University Suzhou Jiangsu China

**Keywords:** acetylation, colorectal cancer, glycolysis, immunotherapy, *N*‐acetyltransferase 1 (NAT1), TNF receptor‐associated factor 6 (TRAF6)

## Abstract

Metabolic dysregulation is a hallmark of tumorigenesis and profoundly impacts immune surveillance; however, the underlying mechanisms and targeted treatments remain limited in colorectal cancer (CRC). Here, we utilize public databases, mouse models, and multi‐omics analyses to identify *N*‐acetyltransferase 1 (NAT1) as a critical prognostic‐associated gene that emerges as a significant modulator of tumor immunity in CRC. We demonstrate that NAT1 suppresses glycolysis and lactate production, thereby promoting an immune‐activated tumor microenvironment (TME). Mechanistically, NAT1 interacts with enolase 1 (ENO1), a key glycolytic enzyme, acetylating it at lysine 343 (K343) and thereby inhibiting its activity. However, loss of NAT1 in tumor cells leads to enhanced ENO1 activation, which in turn drives glycolysis and lactate production. Lactate then binds to TNF receptor‐associated factor 6 (TRAF6), promoting its oligomerization and activation, leading to K63‐linked ubiquitination of PD‐L1, which enhances PD‐L1 stability and facilitates immune evasion. Importantly, low NAT1‐expressing tumors from CRC patients show increased sensitivity to anti‐PD‐1 therapy, and NAT1 deficiency significantly improves the efficacy of anti‐PD‐L1 antibody treatment in preclinical mouse models. Collectively, our findings highlight the critical role of NAT1 in reshaping the TME and suggest that targeting the NAT1‐ENO1‐lactate axis represents a promising therapeutic strategy to enhance immunotherapy in CRC.

## Graphical abstract

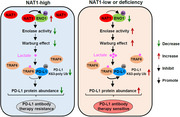

## 1 Introduction

Colorectal cancer (CRC) remains a major global health burden, characterized by persistently high incidence and mortality rates [1, 2]. Its initiation and progression are driven by complex genetic and epigenetic alterations [3, 4] and tumor–immune interactions [5], collectively fostering tumor heterogeneity and limiting therapeutic options—including immunotherapy. Although immune checkpoint blockade (ICB) has demonstrated remarkable efficacy in multiple malignancies [6], it remains largely ineffective in many CRC patients. This resistance stems from low tumor immunogenicity and an immunosuppressive tumor microenvironment (TME) [7].

Emerging evidence indicates that programmed death ligand 1 (PD‐L1; also termed CD274) is abundantly expressed in various cancers, including CRC [8], where it drives immune evasion by binding programmed cell death protein 1 (PD‐1) on activated T cells, thereby inducing T‐cell exhaustion [9, 10]. Recent studies have revealed that several molecules modulate PD‐L1 expression; for example, circRHBDD1 upregulates PD‐L1 to promote immune escape [11]. While elevated PD‐L1 expression may predict improved clinical responses to PD‐1/PD‐L1 blockade [12], the regulatory mechanisms governing its expression in CRC remain incompletely elucidated.

Metabolic reprogramming is a hallmark of tumor cells, characterized by increased lactate secretion that promotes tumor progression [13]. Lactate metabolism in tumor cells has been linked to immunotherapy resistance [14, 15]. Tumor‐derived lactate is taken up by CD8^+^ T cells, suppressing their activation and impairing antitumor immune responses [16, 17]. In addition, tumor cells themselves can utilize lactate to induce PD‐L1 transcription, which inhibits T‐cell antitumor activity [18]. However, the regulatory mechanisms governing lactate production in CRC require further exploration, and crucially, how lactate within tumor cells is sensed as a signaling molecule to regulate PD‐L1 protein stability remains unclear.

In this study, we employed public databases, mouse models, and multi‐omics analyses to identify NAT1 as the key prognosis‐related gene, revealing its role as a potent modulator of tumor immunity in CRC. We found that low NAT1 expression correlates with poor prognosis in CRC patients, and NAT1 loss promotes an immunosuppressive TME. We revealed that NAT1 loss in tumor cells reduces acetylation of ENO1 at K343, enhancing ENO1 activity and driving elevated glycolysis and lactate accumulation. We further demonstrate that TRAF6 functions as an intracellular lactate sensor essential for lactate‐mediated stabilization of PD‐L1 protein, thereby promoting immune evasion. Our findings elucidate how tumor cell‐intrinsic NAT1 governs immune escape during CRC tumorigenesis, providing a therapeutic rationale for targeting this pathway in NAT1‐low or deficient CRC patients.

## 2 Results

### 2.1 NAT1 Deficiency Predicts Aggressive Phenotype, Poor Prognosis, and an Immunosuppressive Microenvironment in CRC

**FIGURE 1:**
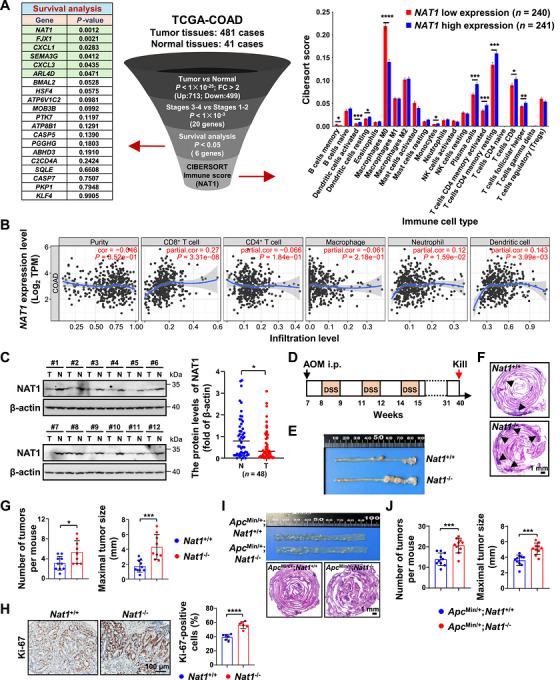
NAT1 deficiency predicts an aggressive phenotype and poor prognosis in colorectal cancer (CRC), promoting tumorigenesis in mouse models. (A) A computational workflow demonstrating our identification of key regulatory molecules associated not only with tumor progression and prognosis but also with tumor immunity. (B) Analysis of the correlation between *NAT1* mRNA expression and tumor cell purity, alongside the infiltration levels of CD8^+^, CD4^+^, macrophages, neutrophils, and dendritic cells in COAD, using TIMER 2.0 (https://compbio.cn/timer2/). (C) Protein expression levels of NAT1 in select pairs of CRC tumors (T) and matched adjacent normal tissues (N). Twelve randomly selected pairs of CRC tumors are shown (left), with quantification of NAT1 expression levels presented (*n* = 48) (right). (D) Experimental workflow for the AOM/DSS‐induced colon cancer model. (E and F) Representative photographs (E) and H&E‐stained sections (F) of colons from AOM/DSS‐induced *Nat1* ^+/+^ (*n* = 10) and *Nat1* ^−/−^ (*n* = 9) mice. (G) Quantification of tumor numbers (left) and tumor sizes (right) across the indicated genotypes. (H) Representative Ki‐67 staining of colon tumors from *Nat1* ^+/+^ and *Nat1* ^−/−^ mice (left); the percentage of tumor cells exhibiting Ki‐67 positive nuclear immunostaining in the colon tumors from both genotypes (right) (*n* = 6 per group). (I) Representative photographs (top) and H&E‐stained sections (bottom) of intestines from *Apc* ^Min/+^; *Nat1* ^+/+^ and *Apc* ^Min/+^; *Nat1* ^−/−^ mice (*n* = 10 per group). (J) Quantification of intestinal tumor numbers (left) and tumor sizes (right) across the indicated genotypes. Data are presented as mean ± SD. *p* values were determined by a two‐tailed Student's *t*‐test (A,C,G,H,J). ^*^ *p* < 0.05, ^**^ *p* < 0.01, ^***^ *p* < 0.001, ^****^ *p* < 0.0001.

To identify key regulatory molecules associated not only with tumor progression and prognosis but also with tumor immunity, we analyzed the RNA‐seq dataset from The Cancer Genome Atlas Colon Adenocarcinoma (TCGA‐COAD), comprising 481 tumor tissues and 41 normal tissues (Figure 1A). Our systematic investigation began with the identification of differentially expressed genes (DEGs), revealing 713 upregulated and 499 downregulated genes in tumor tissues relative to normal controls (Figure S1A). To assess their involvement in tumor progression, we further examined the expression profiles and identified 20 genes exhibiting significant differential expression (*p* < 1 × 10^−3^) (Figure S1B). Subsequent survival analysis using Kaplan–Meier curves pinpointed six genes significantly associated with patient prognosis (*p* < 0.05) (Figure 1A, left). Among these, *N*‐acetyltransferase 1 (NAT1) emerged as the most prominent prognostic marker, with lower expression correlating strongly with poorer overall survival (Figure 1A, left and Figure S1C).

To elucidate the immune contexture associated with these prognosis‐related genes, we applied the CIBERSORT algorithm to evaluate immune cell infiltration in the TME. This analysis revealed that *NAT1* expression was significantly correlated with immune infiltration, particularly T‐cell subsets, exceeding the associations observed for other candidate genes (Figure 1A, right and Figure S1D). Consistently, data from the Tumor Immune Estimation Resource 2.0 (TIMER 2.0) revealed a weak positive correlation between *NAT1* mRNA levels and CD8^+^ T‐cell infiltration in COAD samples, while showing little to no correlation with other immune cell types (Figure 1B). Furthermore, our analysis validated significant downregulation of NAT1 expression in CRC tumor tissues compared to adjacent normal tissues (Figure 1C and Figure S1E). Collectively, these findings highlight NAT1 as a critical prognostic biomarker and a potential key modulator of tumor immunity in CRC.

### 2.2 NAT1 Deficiency Promotes Colorectal Tumorigenesis in AOM/DSS and *Apc*‐Mutant‐Induced CRC Mouse Models

To elucidate the in vivo functions of NAT1 in the development of CRC, we established an Azoxymethane/Dextran Sodium Sulfate (AOM/DSS)‐induced colon cancer model in *Nat1* ^+/+^ (wild‐type) and *Nat1* ^−/−^ (knockout) mice (Figure 1D). Our results indicated that *Nat1* ^−/−^ mice exhibited a greater number of visible colon tumor foci and a significant increase in both tumor numbers and burden (Figure 1E–G). *Nat1* deficiency also significantly enhanced tumor cell proliferation, as assessed by Ki‐67 analysis in colon tumor samples (Figure 1H). In line with these findings, the deletion of *Nat1* in an *Apc*‐mutant‐driven mouse model led to larger intestinal tumor foci (Figure 1I), an increased total number of tumors, and a greater maximum tumor size (Figure 1J), as well as enhanced tumor cell proliferation (Figure S1F). These in vivo data collectively demonstrate that NAT1 acts as a tumor suppressor in CRC pathogenesis.

### 2.3 NAT1 Loss Reshapes the Immune TME and Impairs CD8^+^ T‐Cell Antitumor Function

**FIGURE 2:**
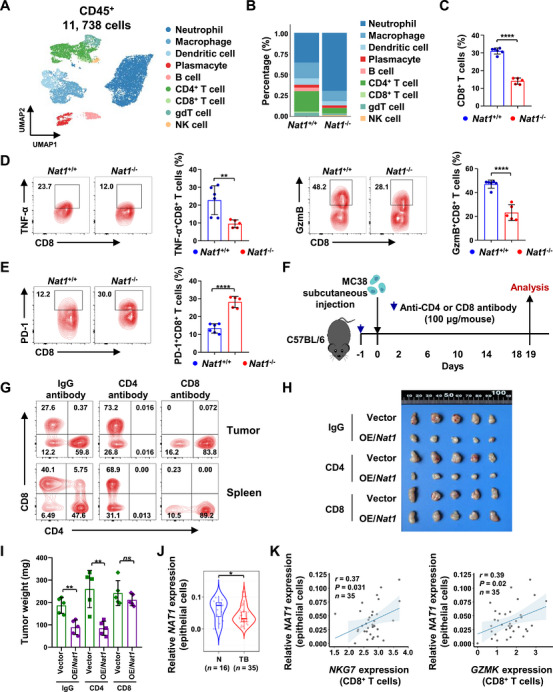
Loss of NAT1 alters the tumor microenvironment (TME) and impairs CD8^+^ T‐cell‐mediated antitumor activity. (A) UMAP plots illustrating scRNA‐seq cluster visualizations of colon tumors from AOM/DSS‐induced *Nat1* ^+/+^ and *Nat1* ^−/−^ mice. (B) Proportions of each cell cluster across the different groups. (C–E) Flow cytometry analyses demonstrating the percentages of CD8^+^ T cells (C), TNF‐α^+^CD8^+^ T cells, and GzmB^+^CD8^+^ T cells (D), as well as PD‐1^+^CD8^+^ T cells (E) in colon tumor tissues from AOM/DSS‐induced *Nat1* ^+/+^ (*n* = 6) and *Nat1* ^−/−^ (*n* = 5) mice. (F) Schematic representation of the experimental procedure for in vivo depletion of CD4^+^ or CD8^+^ T cells. (G) Assessment of CD8^+^ and CD4^+^ T cells by flow cytometry in tumors and spleens of C57BL/6 mice following treatment with IgG, anti‐CD4, or anti‐CD8 depleting antibodies. (H and I) Tumor images (H) along with their corresponding tumor weights (I) (*n* = 5 per group). (J) Violin plots comparing the expression distributions of *NAT1* in the intestinal epithelial cells of both core and border tumors (TB) (*n* = 35) relative to adjacent normal mucosa (N) (*n* = 16). (K) Scatterplots illustrating the Pearson's correlation between epithelial *NAT1* expression and the levels of cytotoxic genes *GZMK* and *NKG7* in tumor‐infiltrating CD8^+^ T cells from 35 core and border tumors. Data are presented as mean ± SD. *p* values were determined by a two‐tailed Student's *t*‐test (C–E,I,J) or Pearson's correlation analysis (K). ^*^ *p* < 0.05, ^**^ *p* < 0.01, ^****^ *p* < 0.0001; ns, not significant.

To gain a better understanding of the role of NAT1 in the TME, we performed 10× single‐cell RNA sequencing (scRNA‐seq) on colon tumors derived from AOM/DSS‐induced *Nat1* ^+/+^ and *Nat1* ^−/−^ mice, generating high‐resolution single‐cell transcriptomes. Dimensionality reduction using Uniform Manifold Approximation and Projection (UMAP) revealed nine distinct cellular clusters, comprising 11,738 CD45^+^ cells (Figure 2A). These clusters included neutrophils, macrophages, dendritic cells (DCs), plasmacytes, B cells, CD4^+^ T cells, CD8^+^ T cells, gamma‐delta T cells (gdT cells), and natural killer (NK) cells, classified according to established marker gene expression (Figure S2A). Notably, populations of antitumor immune cells, including CD8^+^ T cells, CD4^+^ T cells, and B cells, were significantly reduced in *Nat1* ^−/−^ tumors (Figure 2B). Given the direct role of CD8^+^ T cells in antitumor immunity [19], we next focused on this subset in our study. Consistent with these findings, flow cytometry validated a significant decrease in infiltrating CD8^+^ T cells in *Nat1*‐deficient intestinal tumors (Figure 2C and Figure S2B). Furthermore, these tumor‐infiltrating CD8^+^ T cells displayed a markedly lower expression of cytotoxic effector molecules TNF‐α and granzyme B (GzmB) (Figure 2D), alongside an increased abundance of exhausted PD‐1^+^CD8^+^ T cells (Figure 2E) compared to those in *Nat1*‐normal tumors. Collectively, these data highlight the critical role of NAT1 in modulating the TME.

To assess the impact of NAT1 on CRC tumor growth in vivo, we subcutaneously implanted *Nat1*‐deficient or control MC38 cells into the flanks of immunodeficient NOD‐Scid mice and immunocompetent C57BL/6 mice. *Nat1* silencing modestly enhanced tumor growth in immunodeficient NOD‐Scid mice (Figure S2C). However, in immunocompetent C57BL/6 mice, *Nat1* knockdown resulted in a pronounced acceleration of tumor growth (Figure S2D), suggesting that NAT1 likely functions as a tumor suppressor in CRC by modulating antitumor immunity. Supporting this notion, tumors with *Nat1* silencing also showed significantly reduced CD8^+^ T‐cell infiltration (Figure S2E), with these CD8^+^ T cells displaying markedly lower expression of TNF‐α and GzmB (Figure S2F) and an increased abundance of exhausted PD‐1^+^CD8^+^ T cells (Figure S2G). Conversely, tumors generated from *Nat1‐*overexpressing MC38 cells exhibited slowed tumor growth (Figure S2H), accompanied by increased CD8^+^ T‐cell infiltration and enhanced effector function, as evidenced by lower PD‐1 expression and higher TNF‐α and GzmB expression within these T cells (Figure S2I–K).

To validate the role of specific T‐cell subsets in mediating NAT1‐dependent tumor growth control, we depleted either CD8^+^ or CD4^+^ T cells in immunocompetent C57BL/6 mice bearing subcutaneous tumors derived from *Nat1*‐overexpressing (OE/*Nat1*) or control MC38 cells (Figure 2F). Efficient depletion of T‐cell subsets in tumors and spleens was confirmed by flow cytometry (Figure 2G). Strikingly, depletion of CD8^+^ T cells, but not CD4^+^ T cells, abolished the tumor growth inhibition driven by *Nat1* overexpression (Figure 2H,I). These results highlight that NAT1‐mediated tumor suppression is dependent on CD8^+^ T cells.

Importantly, analogous observations were made in clinical CRC tumor samples [20]. scRNA‐seq data revealed significantly lower *NAT1* expression in the intestinal epithelial cells of both core and border tumors (TB) compared to adjacent normal mucosa (N) (Figure 2J). Furthermore, intestinal epithelial *NAT1* expression positively correlated with the levels of cytotoxic genes *GZMK* and *NKG7* in tumor‐infiltrating CD8^+^ T cells (Figure 2K). These findings reinforce the idea that intestinal epithelial NAT1 plays a crucial role in reshaping the immune TME, particularly by influencing the functionality of CD8^+^ T cells.

### 2.4 NAT1 Interacts With and Acetylates ENO1 at K343 to Inhibit ENO1 Activity and Lactate Production

**FIGURE 3:**
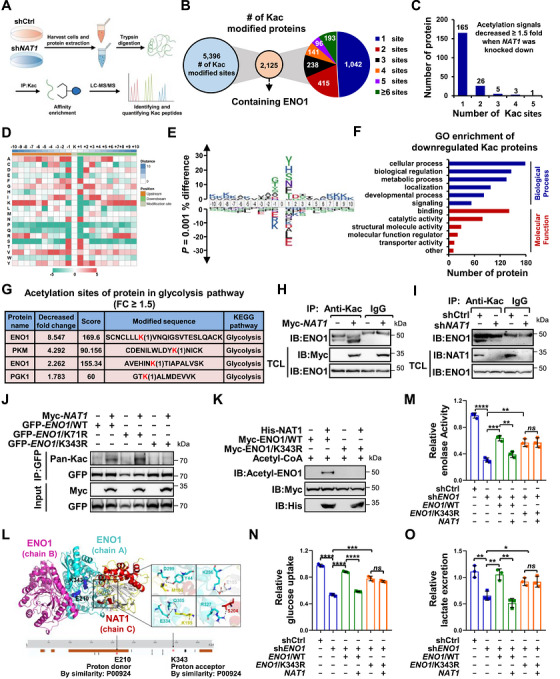
NAT1 interacts with and acetylates ENO1, inhibiting its enzymatic activity and subsequently leading to glycolysis inhibition in CRC. (A) A diagram illustrating the identification of acetyl‐lysine (Kac)‐modified peptides using mass spectrometry (MS). (B) The number of identified Kac sites and Kac‐modified proteins derived from MS (left), accompanied by a histogram showing the distribution of Kac sites per protein (right). (C) Distribution of decreased acetylation sites observed when *NAT1* was knocked down in DLD‐1 cells. (D) A heatmap representing the amino acids surrounding the acetylation sites. (E) Sequence logo of Kac sites identified via MS, generated using pLogo. (F) Pathway enrichment analysis of downregulated Kac proteins was conducted using Gene Ontology (GO) enrichment. (G) Acetylation sites of proteins within the glycolysis pathway. (H and I) WB analysis of total cell lysates (TCLs) and proteins immunoprecipitated with anti‐Kac antibodies from SW480 cells, with or without *NAT1* overexpression (H), or DLD‐1 cells with stable *NAT1* silencing compared to vector control (I). (J) Lysine 343 (K343) is identified as a key acetylation site on ENO1. RKO cells were transfected with the indicated constructs, and whole‐cell lysates were subjected to immunoprecipitation using an anti‐GFP‐tag antibody. WB analysis with an anti‐Kac antibody was performed to detect ENO1 acetylation. (K) In vitro acetylation assays were conducted using purified Myc‐ENO1 or Myc‐ENO1/K343R proteins with purified His‐NAT1 proteins. (L) Structural modeling of the interaction between NAT1 and ENO1, highlighting important interfacial residue contacts. In the ENO1 homodimer, chains A and B are colored cyan and magenta, respectively. NAT1 is represented according to its secondary structure: α‐helix in red, β‐sheet in gray, and loops in yellow. E210 and K343, marked by blue spheres, are crucial residues mediating ENO1‐catalyzed glycolysis. Important interfacial residues are depicted as sticks and labeled in detail. Hydrogen bond interactions are indicated by black dashed lines. (M–O) ELISA analysis of enolase activity (M), glucose uptake (N), and lactate excretion (O) in SW480 cells transfected with the specified plasmids (*n* = 3 per group). Data are presented as mean ± SD. *p* values were determined using one‐way ANOVA with Tukey's post hoc test (M–O). ^*^ *p* < 0.05, ^**^ *p* < 0.01, ^***^ *p* < 0.001, ^****^ *p* < 0.0001; ns, not significant.

Given that NAT1 functions as an acetyltransferase [21], we performed acetylation proteomic analysis to identify lysine‐acetylated (Kac) proteins and sites regulated by NAT1 in shCtrl and sh*NAT1* CRC cells (Figure 3A). In total, we identified 5396 lysine acetylation sites across 2125 proteins. Among these, 1042 proteins (49.0%) contained a single Kac site, while 193 proteins (9.08%) harbored more than six Kac sites (Figure 3B). Notably, 249 Kac sites on 200 proteins were significantly downregulated (fold change ≥ 1.5) upon *NAT1* knockdown (Figure 3C). Sequence analysis of the amino acid residues flanking the Kac sites, compared to the human proteome background, revealed enrichment of alanine (A), lysine (K), and valine (V) residues both upstream and downstream of the acetylated lysines, indicating a preferred local sequence context. Conversely, proline (P) and serine (S) were generally underrepresented at most positions, although serine exhibited modest enrichment at the −1 and +1 positions. In addition, tyrosine (Y) and glycine (G) were highly enriched at the +1 and −1 positions, respectively, while glutamic acid (E) and leucine (L) showed lower but notable enrichment at the +1 position (Figure 3D,E). Functional enrichment analysis indicated that acetylated proteins are predominantly involved in cellular processes, biological regulation, metabolic processes, and signaling pathways (Figure 3F). Importantly, several key glycolytic enzymes, including ENO1, PKM, and PGK1, were significantly acetylated. Among these, acetylation of ENO1 at lysine 343 (K343) was the most prominently downregulated modification following *NAT1* silencing (Figure 3G).

Co‐immunoprecipitation (Co‐IP) assays confirmed that both endogenous and exogenous NAT1 interact with ENO1 (Figure S3A–C). Notably, *NAT1* overexpression robustly enhanced ENO1 acetylation (Figure 3H), while *NAT1* silencing markedly reduced it (Figure 3I). Mass spectrometry (MS) analysis identified two lysine sites, K71 and K343, as potential NAT1 acetylation targets (Figure S3D). Validation using *ENO1* mutants (*ENO1*/K71R and *ENO1*/K343R) revealed that the K343R mutation, but not K71R, substantially reduced acetylation (Figure 3J). In vitro acetylation assays confirmed that NAT1 overexpression increased acetylation in wild‐type ENO1 (ENO1/WT) but not in the ENO1/K343R mutant (Figure 3K). Furthermore, sequence comparisons demonstrated evolutionary conservation of the K343 site across species (Figure S3E). These results establish that NAT1 interacts with and acetylates ENO1 specifically at K343.

Molecular docking also supported the NAT1–ENO1 interaction, revealing hydrogen bonds at the complex interface formed by residues Y44 (ENO1)‐M105 (NAT1), D299 (ENO1)‐M105 (NAT1), K256 (ENO1)‐E155 (NAT1), Q305 (ENO1)‐K195 (NAT1), E334 (ENO1)‐K195 (NAT1), and R327 (ENO1)‐S204 (NAT1). Key catalytic residues E210 and K343 were located within this interaction domain [22] (Figure 3L). NAT1 did not affect *ENO1* mRNA or protein levels (Figure S3F,G). However, *NAT1* overexpression significantly inhibited enolase activity (Figure S3H), while *NAT1* silencing enhanced it (Figure S3I). Based on these observations, we hypothesized that NAT1‐mediated ENO1 acetylation impairs its enzymatic activity. To test this, we depleted endogenous *ENO1* in CRC cells and re‐expressed either *ENO1*/WT or the acetylation‐resistant mutant *ENO1*/K343R. *NAT1* overexpression significantly inhibited the activity of *ENO1*/WT but not *ENO1*/K343R (Figure 3M), demonstrating that K343 is a crucial acetylation site for NAT1's regulation of ENO1 enzymatic activity.

Given ENO1's role as a key glycolytic enzyme [23], we further explored its functional impact on glycolysis. Our results showed that overexpression of *NAT1* significantly decreased critical glycolytic parameters, including glucose uptake and lactate generation (Figure S3J), while silencing *NAT1* had the opposite effect (Figure S3K). Notably, in *ENO1*‐deficient CRC cells, *NAT1* overexpression suppressed glycolytic capacity when *ENO1*/WT was reintroduced, but not when the acetylation‐resistant mutant *ENO1*/K343R was expressed (Figure 3N,O). Together, these findings indicate that NAT1 inhibits ENO1 enzymatic activity and glycolysis through the regulation of ENO1 acetylation.

### 2.5 ENO1 Acetylation at K343 Decreases Lactate Production and Enhances CD8^+^ T‐Cell‐Mediated Antitumor Activity

To assess the functional significance of ENO1 acetylation at K343, we established stable expression of wild‐type *Eno1* (*Eno1*/WT) and the point mutant *Eno1*/K343Q (a lysine‐to‐glutamine mutant that mimics protein hyperacetylation) in MC38 tumor cells (Figure S4A). Our results demonstrated that overexpression of *Eno1*/WT significantly increased enolase activity (Figure S4B), leading to an enhancement in glycolytic output, including glucose uptake and lactate levels (Figure S4C), compared to the control cells. In contrast, overexpression of the acetylation mimic mutant *Eno1*/K343Q did not produce similar effects when compared to *Eno1*/WT (Figure S4B,C). Consistently, metabolic flux analysis via extracellular acidification rate (ECAR) measurement showed that the acetylation mimic mutant *Eno1*/K343Q impaired glycolytic function compared to *Eno1*/WT (Figure S4D). Utilizing ^13^C_6_‐glucose as a tracer for metabolic flux analysis, we also observed a significant decrease in glycolytic intermediates, including phosphoenolpyruvate, pyruvate, and lactate, in *Eno1*/K343Q MC38 cells compared to *Eno1*/WT (Figure S4E). These data support a model in which ENO1 acetylation at K343 suppresses its enzymatic activity, thereby impairing ENO1's role in promoting glycolysis.

To further investigate the role of ENO1 acetylation at K343 in tumor growth, we subcutaneously implanted MC38 cells stably expressing either *Eno1*/WT or *Eno1*/K343Q into immunocompetent C57BL/6 mice. Compared to *Eno1*/WT, *Eno1*/K343Q significantly slowed tumor growth (Figure S4F,G). Flow cytometry analysis demonstrated that tumor‐infiltrating CD8^+^ T cells derived from *Eno1*/K343Q‐expressing tumors exhibited significantly higher expression of the cytotoxic effector molecules TNF‐α and GzmB (Figure S4H,I), along with a reduced frequency of exhausted PD‐1^+^CD8^+^ T cells (Figure S4J), compared to those from *Eno1*/WT‐expressing tumors. These data further demonstrate that ENO1 acetylation at K343 enhances CD8^+^ T cell‐mediated antitumor immunity.

**FIGURE 4:**
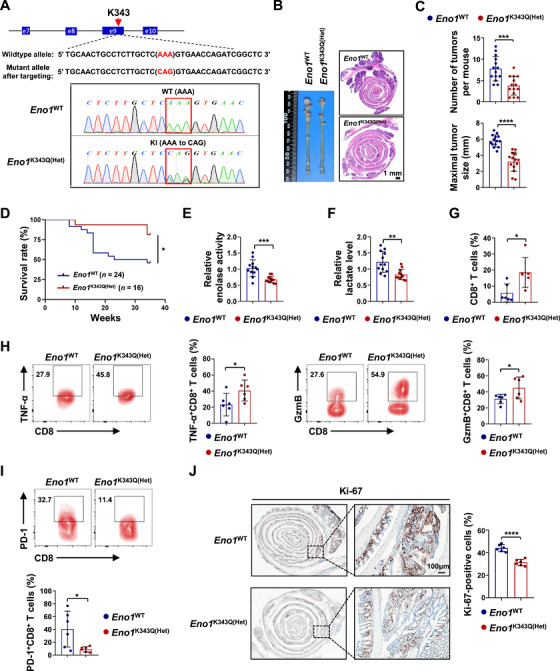
ENO1 acetylation at K343 reduces lactate production and enhances T‐cell antitumor activity in AOM/DSS‐induced *Eno1* ^K343Q(Het)^ mouse model. (A) Overview of the strategy used to generate ENO1 K343Q (*Eno1* ^K343Q^) knock‐in mice via CRISPR‐Cas9 genome editing, followed by sequencing validation. (B and C) Representative images of colons (B, left) and corresponding H&E‐stained sections (B, right), along with quantification of tumor numbers and tumor size (C) in AOM/DSS‐induced *Eno1* ^WT^ and *Eno1* ^K343Q(Het)^ mice (*n* = 14 per group). (D) Kaplan–Meier survival curves for AOM/DSS‐induced *Eno1* ^WT^ (*n* = 24) and *Eno1* ^K343Q(Het)^ mice (*n* = 16). (E and F) ELISA results demonstrating enolase activity (E) and lactate levels (F) in colon tumor tissues from AOM/DSS‐induced *Eno1* ^WT^ and *Eno1* ^K343Q(Het)^ mice (*n* = 12 per group). (G–I) Flow cytometry analysis of the percentages of CD8^+^ T cells (G), TNF‐α^+^CD8^+^ T cells, and GzmB^+^CD8^+^ T cells (H), as well as PD‐1^+^CD8^+^ T cells (I) in colon tumor tissues from AOM/DSS‐induced *Eno1* ^WT^ and *Eno1* ^K343Q(Het)^ mice (*n* = 6 per group). (J) Representative Ki‐67 staining of colon tumors from AOM/DSS‐induced *Eno1* ^WT^ and *Eno1* ^K343Q(Het)^ mice (*n* = 6 per group). Data are presented as mean ± SD. *p* values were determined by a two‐tailed Student's *t*‐test (C,E–J). Survival was analyzed by using the Kaplan–Meier method and compared using the log‐rank test (D). ^*^ *p* < 0.05, ^**^ *p* < 0.01, ^***^ *p* < 0.001, ^****^ *p* < 0.0001.

To validate these findings, we further generated a heterozygous K343Q knock‐in mutation (*Eno1* ^K343Q(Het)^) (Figure 4A), as homozygous K343Q mutations are embryonically lethal. In the AOM/DSS‐induced colon cancer model, we observed significantly reduced tumor growth in *Eno1* ^K343Q(Het)^ mice compared to *Eno1* ^WT^ controls, evidenced by fewer colon tumor foci (Figure 4B) and a lower tumor burden (Figure 4C). Consequently, the acetylation mimic mutant *Eno1*/K343Q mice exhibited extended survival times compared to wild‐type mice (Figure 4D). Furthermore, tumors from *Eno1* ^K343Q(Het)^ mice displayed significantly lower enolase activity (Figure 4E) and reduced lactate levels (Figure 4F). Flow cytometry analysis revealed notable changes in tumor‐infiltrating CD8^+^ T cells of *Eno1* ^K343Q(Het)^ mice, including increased infiltration of CD8^+^ T cells (Figure 4G), and heightened production of TNF‐α and GzmB by CD8^+^ T cells (Figure 4H). In addition, *Eno1* ^K343Q(Het)^ mice exhibited a reduced proportion of exhausted PD‐1^+^CD8^+^ T cells (Figure 4I). The levels of the cell proliferation marker Ki‐67 were also lower in colon tumors from *Eno1* ^K343Q(Het)^ mice compared to those from *Eno1* ^WT^ mice (Figure 4J). Collectively, these findings demonstrate that ENO1 acetylation inhibits enolase activity and lactate production, thereby promoting the formation of an immunoactive TME and suppressing tumor growth.

### 2.6 NAT1 Deficiency Drives Tumor Immune Evasion by Promoting PD‐L1 Expression

**FIGURE 5:**
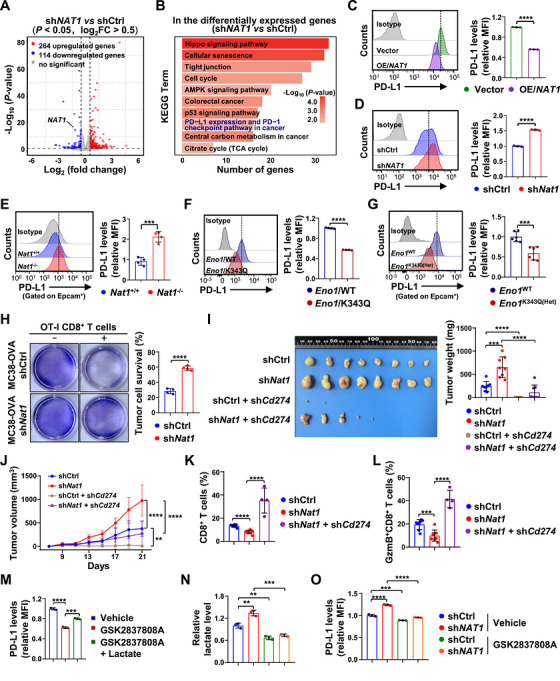
NAT1 regulates PD‐L1 expression in CRC cells. (A) Volcano plot illustrating the differentially expressed genes (DEGs) identified by RNA‐seq analysis in DLD‐1 cells (sh*NAT1* vs. shCtrl). (B) KEGG enrichment analysis of pathways associated with DEGs. (C–G) Histograms (left) and mean fluorescence intensity (MFI) summaries (right) showing PD‐L1 levels in RKO cells with intrinsic overexpression (C) or silencing (D) of *NAT1* (*n* = 3 per group), in the intestinal epithelium from colon tumors of AOM/DSS‐induced *Nat1* ^+/+^ and *Nat1* ^−/−^ mice (*n* = 4 per group) (E), in colon tumor MC38 cells with stably expressed wild‐type *Eno1* (*Eno1*/WT) or mutant *Eno1*/K343Q (*n* = 4 per group) (F), and in the intestinal epithelium from colon tumors of AOM/DSS‐induced *Eno1* ^WT^ and *Eno1* ^K343Q(Het)^ mice (*n* = 6 per group) (G). (H) Naive CD8^+^ T cells were isolated from OT‐I mice and co‐cultured with either *Nat1*‐silenced or control MC38‐OVA cells. These CD8^+^ T cells were subsequently stimulated with anti‐CD3/CD28 antibodies to induce activation. Representative images are shown (left), accompanied by statistical analysis (right) (*n* = 4 per group). (I and J) Representative images of excised tumors (I, left) and their corresponding tumor weights (I, right), along with the tumor growth curve (J) (*n* = 8 per group). (K and L) Flow cytometry analysis showing the percentages of CD8^+^ T cells (K, *n* = 8, 8, 4) and GzmB^+^CD8^+^ T cells (L, *n* = 8, 8, 4) in the indicated groups. (M) SW480 cells were pretreated with GSK2837808A (10 µM, APExBIO, Cat. #B4929) for 6 h before exposure to lactate (0.5 mM, APExBIO, Cat. #M1355) for 15 h, after which PD‐L1 expression was analyzed using flow cytometry (*n* = 3 per group). (N and O) ELISA was used to measure lactate levels (N), and flow cytometry was employed to assess PD‐L1 expression (O) in *NAT1*‐silenced RKO cells treated with or without GSK2837808A (10 µM) for 18 h (*n* = 3 per group). Data are presented as mean ± SD. *p* values were determined by a two‐tailed Student's *t*‐test (C–H), one‐way ANOVA with Tukey's post hoc test (I,K–O), or two‐way ANOVA (J). ^**^ *p* < 0.01, ^***^ *p* < 0.001, ^****^ *p* < 0.0001.

To investigate the underlying mechanism of tumor cell‐intrinsic NAT1 regulation of CD8^+^ T‐cell‐mediated antitumor immunity, we performed transcriptomic profiling of control and *NAT1*‐silenced human DLD‐1 colon cancer cells. Our results indicated that silencing *NAT1* resulted in the upregulation of 264 genes and the downregulation of 114 genes compared to control cells (Figure 5A). Notably, pathway enrichment analysis revealed significant alterations in genes associated with the PD‐L1/PD‐1 checkpoint pathway in cancer (Figure 5B). PD‐L1, the ligand for the PD‐1 immune checkpoint, is commonly upregulated in tumor cells and contributes to immune evasion [24]. In vitro, we observed that overexpression of *NAT1* in both human and mouse CRC cells significantly inhibited PD‐L1 protein expression (Figure 5C and Figure S5A,B), while silencing *NAT1* greatly enhanced PD‐L1 protein expression in these cells (Figure 5D and Figure S5C,D). Notably, NAT1 did not affect the *CD274* mRNA levels (Figure S5E,F), suggesting post‐transcriptional regulation. Consistent with these in vitro findings, intestinal epithelium from colon tumors of *Nat1* ^−/−^ mice in the AOM/DSS model exhibited a significant increase in PD‐L1 protein expression compared to tumors from wild‐type mice (Figure 5E), confirming that NAT1 negatively regulates PD‐L1 protein levels in CRC.

Given the above findings that NAT1 acetylates ENO1 at K343 and inhibits its activity, we next investigated whether NAT1 regulates PD‐L1 through ENO1 acetylation. Overexpression of *Eno1*/WT, but not the acetylation‐mimic mutant *Eno1*/K343Q, promoted PD‐L1 protein expression (Figure 5F). Furthermore, intestinal epithelial cells from AOM/DSS‐treated *Eno1* ^K343Q(Het)^ mice expressed lower PD‐L1 protein levels than those from wild‐type mice (Figure 5G), indicating that ENO1 acetylation at K343 impairs PD‐L1 expression.

Since PD‐L1 suppresses T‐cell function, we performed T cell‐mediated tumor killing assays by co‐culturing activated mouse OT‐I CD8^+^ T cells with MC38‐OVA cells. Silencing *Nat1* in MC38‐OVA cells significantly impaired CD8^+^ T‐cell cytotoxicity (Figure 5H), demonstrating that tumor cell‐intrinsic *Nat1* loss suppresses CD8^+^ T‐cell function. To establish a functional link between PD‐L1 and in vivo tumor growth controlled by NAT1, we deleted the *Cd274* gene in MC38 tumor cells and evaluated their growth alongside the antitumor efficacy of CD8^+^ T cells in immunocompetent C57BL/6 mice. Mice inoculated with sh*Cd274* cells developed significantly smaller tumors than those injected with control cells (Figure 5I). Remarkably, *Cd274* depletion delayed tumor growth driven by *Nat1* loss (Figure 5J). Flow cytometry analysis revealed that *Nat1* deficiency in tumor cells decreased tumor‐infiltrating CD8^+^ T cells and reduced the proportion of cytotoxic GzmB^+^CD8^+^ T cells (Figure 5K,L). Critically, these immunosuppressive effects of *Nat1* deficiency were significantly reversed upon *Cd274* depletion (Figure 5K,L). Therefore, tumor cell‐intrinsic NAT1 deficiency drives tumor immune evasion by upregulating PD‐L1.

### 2.7 NAT1 Regulates PD‐L1 Expression in a Manner That Is Dependent on Lactate

Given that tumor cell‐intrinsic *NAT1* deficiency enhances glycolysis and lactate production, we hypothesized that NAT1 regulates PD‐L1 expression through lactate. To test this hypothesis, we supplemented the culture medium of human colon cancer SW480 cells with exogenous lactate, which significantly upregulated PD‐L1 protein levels (Figure S5G). Next, we inhibited lactate production in CRC cells using the potent and selective LDH inhibitor GSK2837808A, which targets both LDHA and LDHB (Figure S5H). This inhibition resulted in a substantial decrease in PD‐L1 protein expression, while the addition of exogenous lactate restored it (Figure 5M). Similarly, treatment of *NAT1*‐silenced RKO cells with GSK2837808A suppressed lactate production (Figure 5N) and decreased PD‐L1 expression that was induced by the loss of *NAT1* (Figure 5O). Together, these results demonstrate that NAT1 regulates PD‐L1 expression through a lactate‐dependent mechanism.

### 2.8 Lactate Promotes PD‐L1 Protein Stability Through Binding and Activating TRAF6

**FIGURE 6:**
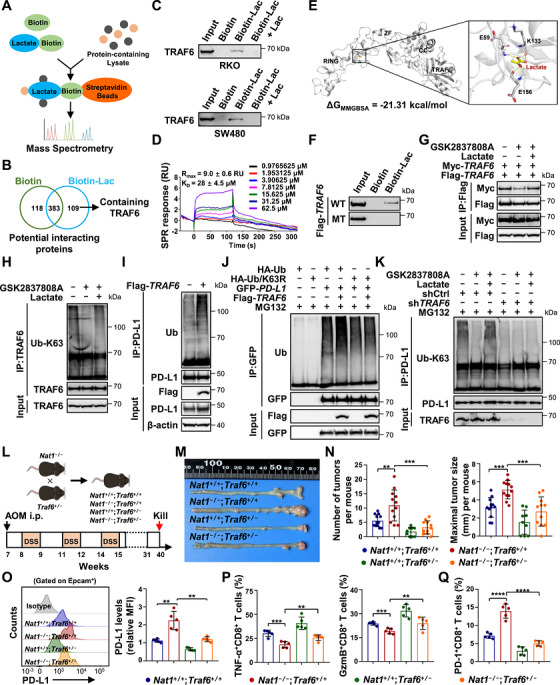
Lactate enhances the stability of PD‐L1 protein by binding to and activating TRAF6. (A) Identification of potential lactate‐interacting proteins. Biotin or biotin‐lactate was incubated with RKO cell lysate, followed by the addition of streptavidin beads, and the complexes were subjected to mass spectrometry analysis. (B) Venn diagram illustrating the number of potential lactate‐interacting proteins. (C) Immunoblotting of binding complexes isolated from the cell extracts of RKO (top) and SW480 (bottom) cells incubated with biotin or biotin‐lactate. (D) An SPR assay validated the interaction between lactate and TRAF6. Biacore T200 evaluation software was used to background subtract and calculate the steady‐state K_D_ value. (E) Binding pose and binding free energy of lactate with TRAF6 following a 1000 ns molecular dynamics simulation. The enlarged view of the binding pocket demonstrates hydrogen bond interactions between lactate and TRAF6 (indicated by yellow dashed lines). (F) Immunoblotting of binding complexes isolated from cell extracts of *TRAF6* or *TRAF6* triple mutant (E59A/K133A/E156A; *TRAF6*‐MT)‐overexpressing SW480 cells incubated with biotin or biotin‐lactate. (G) Immunoprecipitation of Flag‐*TRAF6* followed by immunoblot analysis of Myc‐*TRAF6* in SW480 cells expressing both TRAF6 constructs to assess TRAF6 oligomerization. Cells were pretreated with GSK2837808A (10 µM) for 6 h prior to lactate (0.5 mM) exposure for 6 h. (H) SW480 cells were pretreated with GSK2837808A (10 µM) for 6 h before being exposed to lactate (0.5 mM) for 6 h; immunoprecipitation of endogenous TRAF6 was performed to detect K63‐linked ubiquitination. (I) Immunoprecipitation of endogenous PD‐L1 from *TRAF6*‐overexpressing SW480 cell lysates to assess PD‐L1 ubiquitination. (J) Ubiquitination assays of exogenous PD‐L1 were conducted using lysates from HEK293T cells transfected with HA‐Ub or HA‐Ub/K63R. (K) Ubiquitination assays of endogenous PD‐L1 were performed on lysates from SW480 cells, either with stable *TRAF6* silencing or a control vector, treated with GSK2837808A (10 µM) or lactate (0.5 mM). (L) A schematic illustrating the generation of the indicated mice (top) and the experimental protocol (bottom). (M and N) Representative images of colons (M) and quantifications of colon tumor numbers and tumor burden (N) for the four genotypes (*n* = 12, 12, 9, 11). (O) Histograms (left) and mean fluorescence intensity (MFI) summaries (right) showing PD‐L1 levels in the intestinal epithelium of colon tumors from AOM/DSS‐treated mice of the indicated genotypes (*n* = 5 per group). (P and Q) Flow cytometry analysis of TNF‐α^+^CD8^+^ T cells and GzmB^+^CD8^+^ T cells (P), as well as PD‐1^+^CD8^+^ T cells (Q) in colon tumor tissues from AOM/DSS‐treated mice of the indicated genotypes (*n* = 5 per group). Data are presented as mean ± SD. *p* values were determined using one‐way ANOVA with Tukey's post hoc test (N–Q). ^**^ *p* < 0.01, ^***^ *p* < 0.001, ^****^ *p* < 0.0001.

To elucidate the mechanism by which lactate regulates PD‐L1 expression, we first sought to identify direct lactate sensors. We synthesized biotin‐labeled lactate, incubated it with RKO cell lysates, and performed streptavidin‐bead pull‐down of potential lactate‐interacting proteins, followed by MS analysis (Figure 6A). This identified 109 proteins that potentially interacted with biotin‐lactate but not with biotin alone (Figure 6B). Notably, the E3 ubiquitin ligase TRAF6 emerged as a top hit (Figure 6B and Figure S6A). We confirmed TRAF6–lactate interaction by incubating biotin and biotin‐lactate with lysates from diverse CRC cell lines; this interaction was disrupted by competition with unlabeled lactate (Figure 6C). Surface plasmon resonance (SPR) analysis further confirmed that lactate binds directly to purified recombinant TRAF6 (Figure 6D).

To validate the binding stability and refine the binding pose, we conducted all‐atom molecular dynamics (MD) simulations for 1000 ns. Root mean square deviation (RMSD) analysis showed that TRAF6 adopted a stable conformation between 200 and 350 ns and transitioned to a different stable state after 400 ns (Figure S6B). Root mean square fluctuation (RMSF) analysis indicated that lactate binding enhanced the overall stability of both the coiled‐coil and TRAF6 domains (Figure S6C). Analysis of the binding mode revealed stable hydrogen bonds between lactate and residues E59 and K133 (Figure S6D). Combining these results with binding free energy calculations from the last 100 ns of simulation (Figure S6E), we propose a binding pose for the TRAF6–lactate complex with a binding energy of −21.31 kcal/mol (Figure 6E). These results indicate that residues E59, K133, and E156 are key mediators of lactate binding to TRAF6. Supporting this, the *TRAF6* triple mutant (E59A/K133A/E156A; *TRAF6*‐MT) showed markedly weakened lactate binding compared to wild‐type *TRAF6* (*TRAF6*‐WT) (Figure 6F).

We next explored whether lactate binding influences TRAF6 activity. Our results demonstrated that inhibiting lactate production with GSK2837808A significantly impaired TRAF6 oligomerization. However, the addition of lactate to the culture medium of GSK2837808A‐treated cells markedly restored TRAF6 oligomerization (Figure 6G). TRAF6 dimerization is essential for its K63‐linked auto‐ubiquitination [25]; therefore, the inhibition of lactate production by GSK2837808A led to a decrease in TRAF6 K63‐linked auto‐ubiquitination, which was reversed by the addition of lactate (Figure 6H). These findings suggest that lactate serves as a crucial regulator of TRAF6 activity.

Furthermore, our findings revealed that ectopic expression of *TRAF6* led to an increase in PD‐L1 protein levels (Figure S6F), whereas silencing *TRAF6* significantly reduced PD‐L1 protein levels (Figure S6G) in CRC cells. Consistently, immunofluorescent staining showed that *TRAF6* silencing significantly impaired membrane localization of PD‐L1 (Figure S6H), while overexpression of wild‐type *TRAF6* (*TRAF6*/WT) greatly enhanced the membrane localization of PD‐L1 (Figure S6I). In contrast, the E3 ligase inactive mutant *TRAF6*/C70A [26, 27] failed to upregulate PD‐L1 membrane localization compared to *TRAF6*/WT (Figure S6I), indicating that the E3 ligase activity of TRAF6 is required for regulating PD‐L1 protein expression. In addition, ectopic expression of *TRAF6* markedly enhanced the ubiquitination of PD‐L1 in CRC cells (Figure 6I). Interestingly, the lysine‐deficient ubiquitin mutant (Ub/K63R), which is incapable of forming K63‐linked polyubiquitin chains, did not support TRAF6‐induced ubiquitination of PD‐L1 (Figure 6J), suggesting that TRAF6 mediates the K63‐linked ubiquitination of PD‐L1. Moreover, confocal microscopy revealed colocalization of TRAF6 and PD‐L1 at the membrane (Figure S6J). These findings are consistent with recent observations indicating that TRAF6 positively regulates PD‐L1 at the post‐translational level through their interaction in human embryonic kidney 293 cells [27]; however, it remains unclear whether lactate promotes PD‐L1 protein expression through TRAF6 in CRC. To investigate this, we knocked down *TRAF6* using shRNA in SW480 cells and found that lactate upregulated PD‐L1 protein levels; however, this effect was significantly diminished upon *TRAF6* depletion (Figure S6K). In addition, inhibiting lactate production with GSK2837808A severely impaired K63‐linked ubiquitination of PD‐L1, but lactate supplementation notably restored this modification. Importantly, these effects did not occur when *TRAF6* was silenced (Figure 6K). Collectively, these results indicate that lactate enhances PD‐L1 protein stability by binding to and activating TRAF6.

### 2.9 Reduced *Traf6* Expression Inhibits *Nat1* Deficiency‐Induced PD‐L1 Expression and Enhances T‐Cell Antitumor Immunity in a Mouse Model

To further investigate TRAF6's critical role in *Nat1* deficiency‐induced PD‐L1 expression and colorectal tumorigenesis, we bred *Nat1* ^−/−^ mice with *Traf6* ^+/−^ mice to produce *Nat1* ^−/−^;*Traf6* ^+/−^ offspring (Figure 6L, top). *Traf6* homozygous mice (*Traf6* ^−/−^) appear normal at birth but exhibit poor growth and die prematurely [28]. Therefore, we used *Traf6* heterozygous mice (*Traf6* ^+/−^) and their wild‐type littermates (*Traf6* ^+/+^) to evaluate the role of TRAF6 in vivo. In the AOM/DSS model for colon cancer induction (Figure 6L, bottom), we observed significantly reduced tumor growth in *Traf6* ^+/−^ mice compared to *Traf6* ^+/+^ controls. As shown in Figure 6M,N, *Nat1* ^−/−^;*Traf6* ^+/+^ mice exhibited a marked increase in both the number and size of colon tumors compared to *Nat1* ^+/+^;*Traf6* ^+/+^ mice. Notably, the downregulation of *Traf6* expression in *Nat1* ^−/−^;*Traf6* ^+/−^ mice appeared to partially alleviate these effects. Importantly, *Nat1* loss significantly promoted PD‐L1 expression in the intestinal epithelial cells of colon tumor tissues from *Nat1* ^−/−^;*Traf6* ^+/+^ mice compared to their *Nat1* ^+/+^;*Traf6* ^+/+^ counterparts. In contrast, under the condition of *Nat1* deficiency, additional heterozygous *Traf6* deletion in *Nat1^−/−^;Traf6^+/−^* mice resulted in a substantial reduction of PD‐L1 expression compared to *Nat1^−/−^;Traf6^+/+^* mice (Figure 6O). Next, we examined whether TRAF6 influences T‐cell‐mediated antitumor immunity. In *Nat1*‐deficient tumors, we observed impaired CD8^+^ T‐cell effector function, as evidenced by decreased TNF‐α and GzmB expression (Figure 6P), along with increased populations of exhausted PD‐1^+^CD8^+^ T cells (Figure 6Q). Strikingly, these immune defects were reversed upon *Traf6* downregulation in *Nat1* ^−/−^;*Traf6* ^+/−^ mice (Figure 6P,Q). In conclusion, these findings suggest that TRAF6 is a key molecular driver of immune suppression in NAT1‐deficient colorectal tumors.

### 2.10 NAT1‐Deficient Tumors Display Heightened Sensitivity to Anti‐PD‐L1 Therapy in Preclinical Models

**FIGURE 7:**
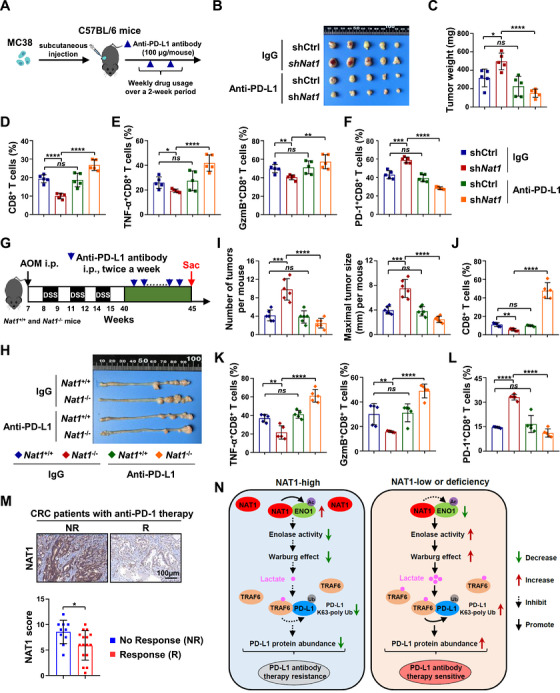
NAT1 deficiency enhances the efficacy of anti‐PD‐L1 therapy in syngeneic and AOM/DSS‐induced CRC models. (A) Schematic representation of the experimental strategy for anti‐PD‐L1 therapy in the MC38 tumor model. (B and C) Tumor images (B) alongside their corresponding tumor weights (C) (*n* = 5 per group). (D–F) Flow cytometry analysis revealing the percentages of CD8^+^ T cells (D), TNF‐α^+^CD8^+^ T cells, and GzmB^+^CD8^+^ T cells (E), as well as PD‐1^+^CD8^+^ T cells (F) within the MC38 tumor (*n* = 5 per group). (G) Diagram illustrating the experimental setup for evaluating anti‐PD‐L1 therapy in the specified mice. (H and I) Representative images of colons (H) and quantification of colon tumor numbers and tumor burden (I) (*n* = 6 per group). (J–L) Flow cytometry analysis showing the percentages of CD8^+^ T cells (J), TNF‐α^+^CD8^+^ T cells, and GzmB^+^CD8^+^ T cells (K), as well as PD‐1^+^CD8^+^ T cells (L) in colon tumor tissues from AOM/DSS‐induced mice (*n* = 5 per group). (M) Relative expression of NAT1 in CRC cells from anti‐PD‐1‐treated CRC patients, assessed by immunohistochemical staining, comparing responders (R, *n* = 16) and nonresponders (NR, *n* = 10). (N) A schematic illustrating the proposed mechanism of NAT1's role in regulating colorectal cancer (CRC). NAT1 downregulates PD‐L1 protein expression by acetylating ENO1, which inhibits lactate‐fueled TRAF6/PD‐L1 signaling (left). Conversely, NAT1 deficiency in CRC cells enhances ENO1 activation, leading to increased glycolysis and lactate production. The lactate then binds to TRAF6, promoting its oligomerization and activation. This results in K63‐linked ubiquitination of PD‐L1, which enhances PD‐L1 protein stability and increases sensitivity to anti‐PD‐L1 immunotherapy (right). Data are presented as mean ± SD. *p* values were determined using one‐way ANOVA with Tukey's post hoc test (C–F,I–L), or two‐tailed Student's *t*‐test (M). ^*^ *p* < 0.05, ^**^ *p* < 0.01, ^***^ *p* < 0.001, ^****^ *p* < 0.0001; ns, not significant.

Building on our observation that NAT1 deficiency drives PD‐L1 induction and CD8^+^ T‐cell exhaustion in CRC, we investigated whether NAT1 expression levels influence the antitumor efficacy of PD‐L1 blockade. Using the MC38 tumor model (Figure 7A), we found that silencing *Nat1* accelerated MC38 tumor growth (Figure 7B,C). Anti‐PD‐L1 monotherapy had limited effects on tumor growth in mice bearing wild‐type (shCtrl) MC38 cells. In contrast, anti‐PD‐L1 treatment significantly reduced both tumor volume and weight in *Nat1*‐silenced MC38 tumors (Figure 7B,C), indicating that *Nat1* deficiency in CRC cells enhances the efficacy of anti‐PD‐L1 therapy. This improved responsiveness in *Nat1*‐deficient versus *Nat1*‐proficient tumors correlated with dramatically increased CD8^+^ T‐cell infiltration (Figure 7D), elevated TNF‐α and GzmB production by tumor‐infiltrating CD8^+^ T cells in response to therapy (Figure 7E), and a reduced percentage of PD‐1^+^CD8^+^ T cells (Figure 7F).

To further validate the therapeutic relevance, we assessed anti‐PD‐L1 therapy in the AOM/DSS‐induced CRC model using *Nat1* ^+/+^ and *Nat1* ^−/−^ mice (Figure 7G). Consistent with our earlier findings, *Nat1* loss significantly enhanced colorectal tumor growth and burden (Figure 7H,I). While anti‐PD‐L1 therapy did not affect tumor growth or burden in wild‐type (*Nat1* ^+/+^) mice, *Nat1* deficiency markedly improved tumor responsiveness to anti‐PD‐L1 therapy (Figure 7H,I). Immunophenotyping revealed that, compared to *Nat1*‐proficient tumors, anti‐PD‐L1 therapy in *Nat1*‐deficient colorectal tumors significantly increased the proportion of tumor‐infiltrating CD8^+^ T cells (Figure 7J), elevated the fractions of TNF‐α^+^CD8^+^ and GzmB^+^CD8^+^ T cells (Figure 7K), and reduced the population of PD‐1^+^CD8^+^ exhausted T cells (Figure 7L). To explore the clinical relevance, we analyzed whether tumoral NAT1 expression correlates with immunotherapy response in CRC patients. Strikingly, low NAT1 expression levels were associated with a significantly better clinical response to anti‐PD‐1 therapy (Figure 7M). Together, these findings demonstrate that low or deficient tumoral NAT1 expression serves as a predictive biomarker for enhanced anti‐PD‐L1 immunotherapy efficacy.

## 3 Discussion

Tumor‐infiltrating lymphocytes (TILs), particularly CD8^+^ T cells, are strongly correlated with improved prognosis and prolonged survival across various cancers, including CRC [29], highlighting their crucial role in tumor control. However, many tumors exhibiting significant T‐cell infiltration continue to progress [30], indicating that endogenous immune responses are often insufficient for achieving tumor clearance. Here, we identify NAT1 as a novel tumor suppressor and immune modulator whose deficiency promotes immunosuppression in the tumor TME via lactate‐mediated stabilization of PD‐L1 protein, ultimately suppressing antitumor T‐cell activity.

NAT1 is a critical Phase II metabolic enzyme that facilitates acetyl‐CoA‐dependent reactions in the metabolism of carcinogens and the regulation of tumor growth [21, 31, 32]. Recent evidence suggests that high NAT1 expression correlates with a favorable prognosis in certain cancers, potentially by inhibiting tumor growth and metastasis [33, 34]. However, the functional role of NAT1 in tumor immunity and its regulatory mechanisms remain unclear. Our findings provide a deeper understanding of how NAT1 influences immune responses, particularly in CRC, where lower expression levels are associated with poor patient prognosis. This correlation underscores NAT1's dual role: it not only impacts tumor growth but also shapes the immune landscape, fostering a TME conducive to immune evasion.

We present a previously unrecognized molecular mechanism through which NAT1 operates as an acetyltransferase that targets the glycolytic enzyme ENO1. NAT1 directly interacts with ENO1, acetylating it specifically at lysine 343 (K343), thus inhibiting its enzymatic activity. The loss of NAT1 leads to hyperactivation of ENO1, resulting in increased glycolysis and lactate production. This lactate then binds to TRAF6, promoting TRAF6 oligomerization and K63‐linked auto‐ubiquitination, which activates TRAF6. The activated TRAF6 subsequently catalyzes K63‐linked polyubiquitination of PD‐L1, preventing its degradation and ultimately suppressing antitumor T‐cell activity (Figure 7N). While K63‐linked ubiquitination is indeed classically associated with signaling rather than proteasomal degradation, emerging evidence indicates that K63 ubiquitination can also influence protein stability by altering trafficking, endocytosis, or proteasomal recognition via indirect mechanisms [35]. Our data suggest that in the case of PD‐L1, TRAF6‐mediated K63 ubiquitination enhances protein stability by promoting membrane localization and reducing turnover through the proteasome pathway.

Recent studies have shown that tumor‐derived lactate suppresses CD8^+^ T‐cell activation and weakens antitumor immune responses [16, 17, 19]. Upon uptake, lactate disrupts pyruvate metabolism and succinate signaling, thereby reducing CD8^+^ T‐cell cytotoxicity [16]. Furthermore, lactate directly binds to GLUT10, impairing glucose transport and the effector functions of T cells [17]. Our recent study identifies tumor‐derived lactate as a natural inhibitor of the extracellular matrix receptor CD44, through which it directly suppresses CD8^+^ T‐cell antitumor activity [19]. Conversely, a recent study suggests that the acidic TME can modulate nanoparticle‐potentiated gastric cancer photoimmunotherapy [36], thus representing a distinct mechanism that may counteract or interact with lactate‐mediated immunosuppression. While recent studies have established that lactate promotes PD‐L1 expression primarily through transcriptional regulation, notably via histone lactylation impacting upstream pathways [18, 37], it remains unclear whether lactate can also function as a signaling molecule perceived by key cellular proteins to directly regulate PD‐L1 protein stability. Here, we further demonstrate that lactate acts as a signaling molecule that binds and activates TRAF6, thereby enhancing PD‐L1 stability through K63‐linked ubiquitination. This mechanism provides new insights into the molecular basis of lactate‐driven immune evasion. The lactate‐mediated activation of TRAF6 reveals a significant expansion of lactate's role in cancer biology. Beyond its traditional characterization as a metabolic waste product, lactate amplifies immune checkpoint expression and stability. TRAF6 functions as a direct intracellular lactate sensor, highlighting the therapeutic potential of targeting metabolic pathways to restore antitumor immunity. Critically, in an AOM/DSS‐induced CRC model, reduced TRAF6 expression suppressed PD‐L1 expression and enhanced T‐cell‐mediated antitumor immunity.

Our study also highlights the clinical implications of NAT1 expression as a predictive biomarker for immunotherapy response. Although total CD8^+^ T‐cell infiltration is reduced in *NAT1‐*deficient tumors, the proportion of exhausted CD8^+^ T cells is markedly increased. This exhausted T‐cell population may enhance sensitivity to anti‐PD‐L1 therapy, as observed in both syngeneic and AOM/DSS‐induced CRC models. Therefore, the relationship between T‐cell abundance and response to checkpoint blockade is not linear; rather, the functional state and the dependency of exhausted T cells on PD‐1 signaling are key determinants. Clinically, low NAT1 expression levels were associated with a significantly better response to anti‐PD‐1 therapy, suggesting that tumoral NAT1 expression is a promising predictive biomarker for response to anti‐PD‐L1/PD‐1 immunotherapy in CRC.

## 4 Limitations of Our Study

While our study highlights a crucial role for tumor cell‐intrinsic NAT1 in orchestrating CD8^+^ T‐cell‐mediated antitumor immunity through the ENO1‐lactate‐TRAF6‐PD‐L1 axis, several limitations should be acknowledged. First, the mechanisms underlying reduced NAT1 expression in clinical CRC remain unclear, warranting further investigation into potential transcriptional or post‐translational regulation. Second, although our findings suggest that tumor cell‐intrinsic NAT1 primarily acts through CD8^+^ T cells, we cannot rule out the possibility that NAT1 may also influence other immune cell populations (e.g., CD4^+^ T cells or B cells) in shaping the TME. Future studies exploring these possibilities will contribute to a more comprehensive understanding of the immunomodulatory functions of tumor cell‐intrinsic NAT1.

## 5 Conclusion

Our study identifies NAT1 as a pivotal modulator of immune dynamics in CRC, orchestrating a metabolic‐immune checkpoint axis that affects tumor progression and response to therapy. The interplay between metabolism and the immune landscape opens new avenues for therapeutic interventions, particularly in targeting immune evasion mechanisms within CRC. By focusing on the vulnerabilities arising from NAT1 deficiency, we can enhance the effectiveness of existing treatments and develop a more robust therapeutic strategy customized to the specific needs of CRC patients.

## 6 Materials and Methods

Detailed Materials and Methods can be found in Supporting Information.

### 6.1 Animal Studies

All mice were housed in a specific pathogen‐free (SPF) facility. The *Nat1* ^−/−^ transgenic mice and *Eno1* ^K343Q^ knock‐in mice were both generated by Cyagen Biosciences Inc. using CRISPR/Cas9‐mediated genome editing. The targeting guide RNA sequences used for the *Eno1* ^K343Q^ model were as follows: gRNA1 (matching the forward strand of the gene), 5′‐CCCTTCCTTAATCAGCAGATGGG‐3′; and gRNA2 (also matching the forward strand), 5′‐GCTGCACCAAGCATGATTGACGG‐3′. The *Apc* ^min/+^ mice were obtained from The Jackson Laboratory. All strains were maintained on a C57BL/6 genetic background. To generate *Apc* ^min/+^;*Nat1* ^−/−^ offspring, *Apc* ^min/+^ mice were subsequently bred with *Nat1* ^−/−^ mice. The AOM/DSS‐induced colon cancer model was developed following a protocol previously optimized in our laboratory [38].

### 6.2 Human Subjects

Human tissue samples were collected in compliance with the ethical principles outlined in the Declaration of Helsinki. Primary tumor tissues from CRC patients, aged 50–80 years, were sourced from The Fourth Affiliated Hospital of Soochow University and The First Affiliated Hospital of Soochow University. Written informed consent was obtained from all participants.

### 6.3 Statistical Analysis

All experiments were performed independently in triplicate. Statistical analysis was performed using GraphPad Prism version 8.0. Results are displayed as mean ± SD. A two‐tailed Student's *t*‐test was used to compare treatments with control groups. One‐way ANOVA with Tukey's post hoc test was applied for multiple comparisons. Statistical significance is represented as follows: *p* < 0.05 (^*^), *p* < 0.01 (^**^), *p* < 0.001 (^***^), *p* < 0.0001 (^****^); ns denotes not significant.

## Author Contributions

H.W. conceptualized the idea and supervised the project. Y.‐X.L., W.‐X.W., Y.W., Y.Y., Z.J., Y.‐Y.W., Y.‐M.H., R.‐Y.Z., H.‐Y.Z., and X.‐S.H. performed biological and mouse experiments and analyzed data. Y.Z., F.L., X.‐M.L., P.‐A.C., and W.‐J.G. collected colorectal cancer samples and analyzed data. X.G., G.H., and Z.‐D.P. performed bioinformatics analysis. Y.‐X.L. and H.W. wrote, reviewed, and edited the manuscript. All authors read and approved the final manuscript.

## Funding

This work was supported by the National Natural Science Foundation of China (82302967, 82372662, and 82373142); the Provincial‐level Talent Program for the National Center of Technology Innovation for Biopharmaceuticals (NCTIB2024JS0101); the Natural Science Foundation of Jiangsu Province (BE2023703 and BG2025055); and the Suzhou Industrial Park Healthcare Talent Support Initiative (YQWS202502). This work was also supported by the Basic Research Program of Jiangsu (BK20255001); the Suzhou International Joint Laboratory for Diagnosis and Treatment of Brain Diseases; and the Priority Academic Program Development of Jiangsu Higher Education Institutions.

## Ethics Statement

All animal experiments were conducted in accordance with the guidelines established by the Animal Care and Use Committee of Soochow University (SUDA20250919A06). The human CRC sample study was approved by the Biomedical Research Ethics Committee of Soochow University (SUDA20241014H04).

## Conflicts of Interest

The authors declare no conflicts of interest.

## Supporting information




**Supporting Figure 1**: Public databases identify NAT1 as a key prognosis‐related gene associated with immunity and the progression of colorectal cancer (CRC).
**Supporting Figure 2**: NAT1 deficiency impairs the anti‐tumor activity of CD8+ T cells and promotes tumor growth.
**Supporting Figure 3**: NAT1 binds to and acetylates ENO1 which inhibits its enzymatic activity and ultimately reduces glycolysis in CRC.
**Supporting Figure 4**: Mimicking protein hyperacetylation at ENO1 K343 inhibits its enzymatic activity and lactate production while enhancing T cell anti‐tumor immunity.
**Supporting Figure 5**: NAT1 regulates PD‐L1 expression.
**Supporting Figure 6**: Lactate binds to and activates TRAF6 to enhance PD‐L1 protein stability.

## Data Availability

All data are included in the manuscript or Supporting Information. Source data are provided with this paper. Reagents will be shared by the corresponding author upon reasonable request.
