# Supplementary material for: Targeting the NAT1‐ENO1‐Lactate Axis Destabilizes PD‐L1 to Reinvigorate Antitumor Immunity in Colorectal Cancer
Source: MedComm (2020). 2026 Jul 16;7(8):e70867. doi: 10.1002/mco2.70867 (PMC13376828; doi:10.1002/mco2.70867)
Supplement: Supplementary file 1 — Supporting Figure 1: Public databases identify NAT1 as a key prognosis‐related gene associated with immunity and the progression of colorectal cancer (CRC). Supporting Figure 2: NAT1 deficiency impairs the anti‐tumor activity of CD8+ T cells and promotes tumor growth. Supporting Figure 3: NAT1 binds to and acetylates ENO1 which inhibits its enzymatic activity and ultimately reduces glycolysis in CRC. Supporting Figure 4: Mimicking protein hyperacetylation at ENO1 K343 inhibits its enzymatic activity and lactate production while enhancing T cell anti‐tumor immunity. Supporting Figure 5: NAT1 regulates PD‐L1 expression. Supporting Figure 6: Lactate binds to and activates TRAF6 to enhance PD‐L1 protein stability. [file MCO2-7-e70867-s001.pdf]

Targeting the NAT1-ENO1-lactate axis destabilizes PD-L1 to reinvigorate antitumor immunity in colorectal cancer

Yi-Xuan Liu[1,#], Wen-Xin Wu[1,#], Yang Zang[2,#], Guang Hu[3,10,#], Xin Guo[1], Yi Wang[1], Yun Yang[1], Zhi Jiang[4,5], Xiu-Ming Li[6], Feng Liu[7], Yue-Yue Wu[1], Zu-Da Pan[1], Yuan-Meng Hu[1], Ruo-Yang Zhang[1], Hai-Yi Zhang[1], Ping-An Chang[8,*], Xiao-Shun He[6,*], Wen-Juan Gan[1,*] and Hua Wu[1,4,9,10,*]

[1]Department of Pathology, The Fourth Affiliated Hospital of Soochow University, Suzhou Medical College of Soochow University, Soochow University, Suzhou, Jiangsu, China.

[2]Medical College, Yangzhou University, Yangzhou, Jiangsu, China.

[3]Department of Bioinformatics, School of Life Science, Soochow University, Suzhou, Jiangsu, China.

[4]National Center of Technology Innovation for Biopharmaceuticals, Suzhou Biomedical Industry Innovation Center, Suzhou, Jiangsu, China.

[5]Department of Biochemistry and Molecular Biology, Suzhou Medical College of Soochow University, Soochow University, Suzhou, Jiangsu, China.

[6]Department of Pathology, The First Affiliated Hospital of Soochow University, Suzhou, Jiangsu, China.

[7]Department of General Surgery, Suzhou Guangci Cancer Hospital, Suzhou, Jiangsu, China.

[8]Department of Urology Surgery, Affiliated Dongtai Hospital of Nantong University, Dongtai, Jiangsu, China.

[9]Cancer Institute, Suzhou Medical College of Soochow University, Soochow university, Suzhou, Jiangsu, China.

[10]Biomedical Basic Research Center (BBRC) of Jiangsu, Soochow University， Suzhou, Jiangsu, China.

[#]These authors contributed equally

Running title: Targeting NAT1 for Colorectal Cancer Immunotherapy

Conflict of interest: None to declare

***Corresponding authors:**

Ping-An Chang, Department of Urology Surgery, Affiliated Dongtai Hospital of Nantong University, China; E-mail: cpacpa01@sina.com

Xiao-Shun He, Department of Pathology, The First Affiliated Hospital of Soochow University, Suzhou, Jiangsu, China; E-mail: sdblhxs@163.com

Wen-Juan Gan, Department of Pathology, The Fourth Affiliated Hospital of Soochow University, China; E-mail: ganwenjuan@suda.edu.cn.

Hua Wu, Ph.D, Professor, Department of Pathology, The Fourth Affiliated Hospital of Soochow University, Suzhou Medical College of Soochow University, Soochow University, China; E-mail: wuhua@suda.edu.cn.

Supplementary Materials and Methods

In Vivo depletion of CD4$^+$ or CD8$^+$ T Cells

To achieve *in vivo* depletion of CD8$^+$ T cells, mice were intraperitoneally injected with 100 μg of anti-CD4 (Selleck, Cat. #A2101) or anti-CD8 antibody (Selleck, Cat. #A2102) one day prior to tumor inoculation, followed by twice-weekly injections throughout the experimental period to maintain consistent depletion of the CD8$^+$ T cell subset. A control group of mice received IgG isotype antibody (Selleck, Cat. #A2116) under the same conditions.

In Vivo treatments

To evaluate the antitumor efficacy of the PD-L1 antibody, AOM/DSS-induced *Nat1*$^{+/+}$ and *Nat1*$^{-/-}$ mice were treated with either anti-IgG (Selleck, Cat. #A2116) or anti-PD-L1 (Selleck, Cat. #A2115) antibodies (10 mg/kg, administered intraperitoneally twice weekly). After the designated treatment period, the mice were euthanized, and their colon tissues were collected for further analysis.

In separate experiments involving PD-L1 antibody treatment in C57BL/6 mice, 1×10^6 MC38 cells, either stably silencing *Nat1* or expressing a control, were injected subcutaneously into the right flanks of the mice. One week after tumor cell injection, the mice were randomly assigned to four subgroups and received either PD-L1 antibody (100 μg per mouse, administered intraperitoneally twice weekly) or an IgG control for two weeks. At the end of the treatment period, the mice were sacrificed, and tumor nodules were excised for further assessment.

Cell culture and transfection

The human colon cancer cell lines SW480, DLD-1, HCT116, RKO, murine colon adenocarcinoma cell line MC38, and human embryonic kidney cell line HEK293T were obtained from the Cell Bank of the Chinese Academy of Sciences (Shanghai,

China). All cell lines were cultured at 37°C in a humidified incubator with 5% CO_2. HEK293T and MC38 cells were maintained in Dulbecco's Modified Eagle Medium (DMEM) supplemented with 10% fetal bovine serum (FBS) and 1% penicillin-streptomycin (Invitrogen). RKO, SW480, DLD-1 and HCT116 cells were cultured in RPMI-1640 medium supplemented with 10% FBS and 1% penicillin-streptomycin, as previously described [1]. All cell lines were authenticated by short tandem repeat (STR) analysis and routinely tested for mycoplasma contamination to ensure purity. Stable cell lines with *NAT1* gene silencing was generated using lentiviral shRNA techniques following established protocols [2]. The targeting sequence for human *NAT1* was TGGCAGCCTCTGGAGTTAATT, the targeting sequence for mouse *Nat1* was GCACATCGTTCTGTTCCTTGC, and the control sequence for both human and mouse constructs was TTCTCCGAACGTGTCACGT. For pharmacological inhibition experiments, cells were treated with the LDHA inhibitor GSK2837808A (APExBIO, Cat. #B4929) or exogenous lactate (APExBIO, Cat. #M1355) at indicated concentrations and durations.

Antibodies and reagents

The antibodies for flow cytometry analysis and $CD8^+$ T cell isolation were purchased from BioLegend, including PerCP anti-mouse CD45 (Cat. #103130, 1:200), BV785 anti-mouse CD3 (Cat. #100232, 1:200), APC anti-mouse CD8α (Cat. #100712, 1:200), APC-Cy7 anti-mouse CD8α (Cat. #100714, 1:200), PE anti-mouse CD4 (Cat. #100408, 1:250), BV421 anti-mouse TNF-α (Cat. #506328, 1:200), APC anti-human/mouse Granzyme B (Cat. #372204, 1:250), PE anti-human/mouse Granzyme B (Cat. #372208, 1:400), PE anti-mouse PD-1 (Cat. #135206, 1:200), BV605 anti-mouse Ep-CAM (Cat. #118227, 1:200), BV605 anti-mouse PD-L1 (Cat.

#124321, 1:200), APC anti-mouse PD-L1 (Cat. #124312, 1:200), APC anti-human PD-L1 (Cat. #329708, 1:400), Zombie Red™ Fixable Viability Kit (Cat. #423110, 1:1,000), and Zombie Aqua™ Fixable Viability Kit (Cat. #423102, 1:1,000). Additionally, the MojoSort™ Mouse $CD8^{+}$ T Cell Isolation Kit (Cat. #480035), purified anti-mouse CD3ε (Cat. #100302), and purified anti-mouse CD28 (Cat. #102102) were obtained from the same supplier. The LIVE/DEAD™ Fixable Violet Dead Cell Stain Kit (Cat. #L34963, 1:1,000) was sourced from Invitrogen. Antibodies for western blot were as follows: Rabbit anti-ENO1 (Cat. #3810, 1:2,000) was purchased from Cell Signaling Technology (Beverly, MA, USA); Mouse anti-Myc-tag (Cat. #9E10, 1:1,000) was purchased from Santa Cruz Biotechnology (Santa Cruz, CA, USA); Rabbit anti-TRAF6 (Cat. #ab33915, 1:2,000), Rabbit anti-NAT1 (Cat. #ab109114, 1:2,000), and Rabbit anti-Ubiquitin (Cat. #ab134953, 1:2,000) were purchased from Abcam (Cambridge, MA, USA); Mouse anti-Flag-tag (Cat. #66008-4-Ig, 1:10,000), Rabbit anti-Flag-tag (Cat. #20543-1-AP, 1:20,000), Mouse anti-His-tag (Cat. #66005-1-Ig, 1:10,000), Mouse anti-beta-Actin (Cat. #66009-1-Ig, 1:20,000), Rabbit anti-NAT1 (Cat. #19188-1-AP, 1:600), Mouse anti-Myc-tag (Cat. #66003-2-Ig, 1:20,000), Mouse anti-GFP-tag (Cat. #66002-2-Ig, 1:20,000), and Mouse anti-PD-L1 (Cat. #66248-1-Ig, 1:2,000) were purchased from Proteintech (Wuhan, Hubei, China); Rabbit anti-PD-L1 (Cat. #GTX104763, 1:2,000), HRP-conjugated Mouse anti-IgG (Cat. #GTX221667-01, 1:10,000), HRP-conjugated Rabbit anti-IgG (Cat. #GTX221666-01, 1:10,000), and Rabbit anti-alpha-Tubulin (Cat. #GTX112141, 1:20,000) were purchased from GeneTex (Irvine, CA, USA). Lipofectamine 2000 and Trizol were purchased from Invitrogen (Carlsbad, CA, USA) and WesternBright ECL reagents were purchased from Advansta (Menlo Park, CA, USA).

Single-cell RNA-seq (scRNA-seq) analysis

The scRNA-seq analysis involved colon tumors from AOM/DSS-induced *Nat1*$^{+/+}$ *and Nat1*$^{-/-}$ mice. Tumors were digested into single cell suspensions. The scRNA-seq libraries were created using the 10× Genomics Chromium Next GEM Single Cell 3′ Reagent Kits v3.1, following the manufacturer's guidelines. Sequencing was carried out on the Illumina NovaSeq X Plus PE150 platform by Annoroad Gene Tech. (Beijing) Co., Ltd. FASTQ files were processed and aligned to the mm10 mouse reference genome using Cell Ranger software (version 9.0.0) from 10x Genomics, with unique molecular identifier (UMI) counts compiled for each barcode. The original expression matrix produced by Cell Ranger was imported into RStudio for further analysis using Seurat (version 5.3.0). To enhance data quality, we utilized the DoubletFinder package (version 2.0.6) to eliminate multiplets and applied the following filtering criteria: 1) Each cell must have a detected gene count between 300 and 5,000; 2) UMI counts for each cell should range from 500 to 30,000; 3) The percentage of mitochondrial genes must be below 10%; 4) The ratio of hemoglobin-associated genes should be less than 5%; 5) Only genes detected in more than three cells were retained. After quality control, we employed the sctransform package (version 0.4.2) for data normalization and scaling, followed by PCA and UMAP dimensionality reductions. Based on cell expression statistics, we executed the FindNeighbors and FindClusters functions to assess cell-to-cell similarity, construct a cell adjacency graph, and achieve cell clustering. Finally, we annotated the cell clusters by analyzing their markers and determined the proportions of each cluster.

Correlation analysis using scRNA data

Human colorectal cancer single-cell RNA sequencing datasets GSE132465 and GSE144735 were obtained from the GEO database. In addition to standard processing

steps, we integrated the samples using the widely adopted R package Harmony. By leveraging Harmony, we effectively minimized batch-to-batch variability among the samples, which enhanced the accuracy of cell clustering and annotation. We extracted *NAT1* expression levels in epithelial cells and *NKG7/GZMK* expression levels in $CD8^+$ T cells from the integrated Seurat object, categorizing them by sample to compute the average values. Each point on the plots represented a sample, and we compared the mean epithelial *NAT1* expression between normal tissue and the combined tumor/tumor margin regions. The observed differences were evaluated using an unpaired Student's *t*-test. Additionally, we utilized the ggpubr package to generate scatter plots, which included calculated correlation coefficients and fitting curves, allowing us to assess the correlation between *NAT1* expression and the lethality of $CD8^+$ T cells.

RNA sequencing (RNA-seq) analysis

Paired-end reads were harvested from Illumina NovaSeq 6000 platform, and were quality controlled by Q30. After adapter-trimming and low-quality reads removing by CUTADAPT software (version 1.9.3), the high-quality clean reads were aligned to the human reference genome (HG19) with HISAT2 software (version 2.0.4). Then, HTSeq software (version 0.9.1) was used to get the raw count, and DESeq2 was used to perform normalization, then differentially expressed mRNAs were identified by *P*-value and fold change. Pathway enrichment analysis was performed based on the differentially expressed mRNAs.

4D label-free quantitative acetylation proteomics analysis

Cells were harvested, and a 4D label-free quantitative acetylation proteomics analysis was performed by Jingjie PTM BioLabs. For protein extraction, cell samples stored at -80°C were thawed, and each sample group received four volumes of lysis buffer (8

M urea, 1% Protease Inhibitor Cocktail, 3 μM TSA, 50 mM NAM), followed by sonication. Remaining debris was removed by centrifugation at 12,000 g for 10 minutes at 4°C. The supernatant was then transferred to a new centrifuge tube, and protein concentration was determined using a BCA kit according to the manufacturer's instructions.

For trypsin digestion, equal amounts of protein from each sample were subjected to enzymatic digestion, with their volume adjusted using lysis buffer for consistency. Trichloroacetic acid (TCA) was slowly added to a final concentration of 20%, mixed by vortexing, and incubated at 4°C for 2 hours. The samples were subsequently centrifuged at 4,500 × g for 5 minutes, and the supernatant was discarded. The remaining precipitate was washed 2–3 times with pre-chilled acetone. After air-drying, the pellet was resuspended in 200 mM TEAB and sonicated to ensure complete dispersion. For overnight digestion, trypsin was added at a 1:50 trypsin-to-protein mass ratio. The sample was then reduced using 5 mM dithiothreitol (DTT) for 30 minutes at 56°C and alkylated with 11 mM iodoacetamide (IAA) for 15 minutes at room temperature in the dark.

To enrich for acetylation modifications, peptides were dissolved in IP buffer (100 mM NaCl, 1 mM EDTA, 50 mM Tris-HCl, 0.5% NP-40, pH 8.0), and the supernatant was transferred to pre-washed acetyl-lysine antibody-conjugated beads (Cat. #PTM-104, PTM Bio, Hangzhou, China). The mixture was incubated overnight at 4 °C with gentle rotation. After incubation, the beads were washed four times with IP buffer and twice with deionized water. Bound peptides were eluted three times with 0.1% trifluoroacetic acid, and the eluates were collected and vacuum-dried. The dried peptides were then desalted using C18 ZipTips according to the manufacturer's protocol, vacuum-dried again, and analyzed via LC-MS/MS.

For LC-MS/MS analysis, peptides were dissolved in solvent A (an aqueous solution containing 0.1% formic acid and 2% acetonitrile) and separated using a NanoElute UPLC system, while solvent B consisted of 0.1% formic acid in 100% acetonitrile. The liquid chromatography gradient was set as follows: 0–44 minutes, 6%–22% B; 44–56 minutes, 22%–30% B; 56–58 minutes, 30%–80% B; and 58–60 minutes at 80% B, with a constant flow rate of 450 nL/min. Separated peptides were ionized via a capillary ion source and analyzed using a timsTOF Pro mass spectrometer (Bruker). The electrospray voltage was set to 2.0 kV, and both precursor ions and their secondary fragments were detected in the high-resolution TOF analyzer. The MS/MS scan range was set from 100 to 1700 m/z, and data acquisition was performed in parallel accumulation–serial fragmentation (PASEF) mode. Each full MS scan was followed by 10 PASEF MS/MS scans of precursor ions with charge states ranging from 0 to 5, with a dynamic exclusion of 30 seconds applied to prevent the repeated acquisition of the same precursor ions

For database searching, the MS/MS data were processed using MaxQuant (version1.6.6.0). Tandem mass spectra were searched against the Homo_sapiens_9606 database (20,366 sequences) concatenated with a reverse decoy database to estimate the false discovery rate (FDR). A contaminant database was also included to eliminate interference from common contaminants. Trypsin/P was specified as the cleavage enzyme, allowing for up to 4 missed cleavages. The minimum peptide length was set to 7 amino acids, and the maximum number of modifications per peptide was set to 5. The precursor ion mass tolerance was set to 20 ppm for both the first and main searches, while the fragment ion mass tolerance was also set to 20 ppm. Carbamidomethylation on cysteine was specified as a fixed modification, while acetylation (protein N-term), oxidation (methionine), and acetylation (lysine) were

designated as variable modifications. The label-free quantification (LFQ) method was applied, with FDR thresholds for both protein and PSM identifications set to 1%.

Flow cytometry

Single-cell suspensions prepared from cells or tumor tissues were sequentially stained with fluorochrome-conjugated antibodies. Dead cells were excluded using the Zombie Red/Aqua Fixable Viability Kit (BioLegend). For cell surface marker staining, cell suspensions were incubated with fluorochrome-conjugated antibodies in PBS supplemented with 2% FBS, followed by fixation with 4% paraformaldehyde. For intracellular marker staining, cells were processed using the Cytofix/Cytoperm Fixation/Permeabilization Kit (BD Biosciences) before incubation with fluorochrome-conjugated antibodies. Multiparametric analysis was performed on a FACSCelesta Flow Cytometer (BD Biosciences), and data were analyzed using FlowJo software (TreeStar).

In vitro acetylation assay

For the acetylation reaction, immunopurified Myc-tagged ENO1/WT or ENO1/K343R (expressed in HEK293T cells) was incubated with purified His-NAT1 (expressed in E. coli bacterial cells) in 40 μL reaction buffer containing 20 mM Tris-HCl (pH 8.0), 100 mM KCl, 20% glycerol, 1 mM dithiothreitol (DTT), and 0.2 mM EDTA, with or without 100 μM acetyl-CoA. After incubation at 30°C for 1 hour, reactions were stopped by adding 10 μL of 5× SDS sample buffer. Target protein acetylation was analyzed by western blot using a pan-acetyl-lysine-specific antibody.

Immunofluorescence microscopy

Immunofluorescence (IF) microscopy was performed on cultured cells following previously published protocols [3]. Images were acquired using a Leica SP8 confocal microscope and analyzed with ImageJ software.

RNA extraction and qPCR analysis

RNA was isolated from cell lines using the Eastep® Super Total RNA Extraction Kit (Promega, Shanghai; Cat. #LS1040) according to the manufacturer’s protocol, which included on-column genomic DNA removal. RNA concentrations were measured using the Nanodrop One spectrophotometer (Thermo Fisher Scientific, Cat. #840-317400). For cDNA synthesis, 4 µg of RNA was reverse transcribed using the RevertAid First Strand cDNA Synthesis Kit (Thermo Fisher Scientific; Cat. #K1622). qPCR was performed with Taq Pro Universal SYBR qPCR Master Mix (Vazyme; Cat. #Q712) on a real-time PCR system (Thermo Fisher Scientific; Cat. #ABI 7500). The thermal cycling conditions were as follows: initial denaturation at 95°C for 15 minutes, followed by 40 cycles of 95°C for 15 seconds and 60°C for 60 seconds. The following primers were used for quantitative real-time PCR: ENO1-forward: 5’-AGAAGTCCTGCAACTGCCTC-3’; ENO1-reverse: 5’-GATGAGACACCATG -ACGCCC-3’; β-actin-forward: 5’-CATGTACGTTGCTATCCAGGC-3’; β-actin-reverse: 5’-CTCCTTAATGTCACGCACGAT-3’.

Metabolic assays

Metabolic parameters were assessed using commercially available kits. Lactic acid levels were measured with the L-Lactate Assay Kit (Colorimetric) (Abcam, Cat. #ab65331), glucose uptake was quantified using the Glucose Assay Kit (Abcam, Cat. #ab102517), and enolase activity was analyzed with the Enolase Activity Assay Kit (Sigma-Aldrich, Cat. #MAK178). All assays were carried out following the manufacturer's instructions.

Immunoprecipitation (IP) and immunoblot (IB)

Cells were lysed in 1 mL of lysis buffer (20 mM Tris-HCl, pH 7.4, 10 mM EDTA, 100 mM NaCl, and 1% IGEPAL), supplemented with protease inhibitors (Roche), for

30 minutes at 4°C. The lysates were centrifuged at 12,000 × g for 15 minutes, and protein concentrations were subsequently measured. Equal amounts of lysates were used for immunoprecipitation. Specific antibodies were added to the lysates, followed by overnight incubation at 4°C with gentle rocking. Protein A/G beads were then added, and the mixture was incubated for an additional 3 hours at 4°C with continuous rocking. The beads were washed three times with washing buffer (20 mM Tris-HCl, pH 7.4, 10 mM EDTA, 150 mM NaCl, and 1% IGEPAL), boiled in 1× SDS loading buffer for 10 minutes, and subjected to SDS–PAGE analysis. Membranes were blocked with 5% milk in TBST buffer, probed with the indicated antibodies, and analyzed via immunoblotting for downstream applications.

Surface plasmon resonance (SPR) analysis

Surface plasmon resonance (SPR) analysis was conducted following an optimized protocol established in lab [4]. The binding kinetics between TRAF6 and lactate were assessed using the BIAcore T-200 instrument with CM5 chips (GE Healthcare) at 25°C. Following the coupling of purified TRAF6 protein to the CM5 chip, lactate binding to TRAF6 was evaluated by injecting it into a TRAF6-immobilized flow cell at various concentrations. The K_D values were determined using BIAcore T-200 evaluation software v2.0 (GE Healthcare).

Structural Modeling of the NAT1-ENO1 Complex

The ENO1 protein structure was extracted from the human alpha-enolase complex (PDB ID: 2PSN, chains A and B). The NAT1 structure was obtained from human N-acetyltransferase 1 (PDB ID: 2IJA). The NAT1-ENO1 complex was modeled using the ZDOCK Server [5]. The top-ranked model was selected based on ZDOCK scores, which indicated potential interactions between the C-terminus of ENO1 and NAT1. Interface residues within the complex were identified using PyMOL.

Molecular simulations

The TRAF6 structure was obtained from the AlphaFold Protein Structure Database, and lactate 3D model was sourced from PubChem. Potential binding pockets were first predicted with Fpocket [6]; lactate was then docked into TRAF6 using AutoDock Vina [7]. 1000ns molecular dynamics (MD) simulations were performed using GROMACS [8] under the Amber99SB-ILDN force field. All simulated systems were solvated in TIP3P water model and neutralized with Na^+/Cl^- ions. The stability of simulation systems was assessed via root mean square deviation (RMSD), root mean square fluctuation (RMSF), and hydrogen bond dynamics. Binding free energy was calculated with the MM/GBSA method implemented in the gmx_MMPBSA tool, and key lactate-interacting residues were pinpointed through hydrogen-bond occupancy and per-residue energy.

References

1 Wu H, Li XM, Wang JR, Gan WJ, Jiang FQ, Liu Y, et al. NUR77 exerts a protective effect against inflammatory bowel disease by negatively regulating the TRAF6/TLR-IL-1R signalling axis. J Pathol. 2016;238(3):457-469.

2 He XS, Ye WL, Zhang YJ, Yang XQ, Liu F, Wang JR, et al. Oncogenic potential of BEST4 in colorectal cancer via activation of PI3K/Akt signaling. Oncogene. 2022;41(8):1166-1177.

3 Ye WL, Huang L, Yang XQ, Wan S, Gan WJ, Yang Y, et al. TRIM21 induces selective autophagic degradation of c-Myc and sensitizes regorafenib therapy in colorectal cancer. Proc Natl Acad Sci U S A. 2024;121(42):e2406936121.

4 Liu YX, Wan S, Yang XQ, Wang Y, Gan WJ, Ye WL, et al. TRIM21 is a druggable target for the treatment of metastatic colorectal cancer through ubiquitination and activation of MST2. Cell Chem Biol. 2023;30(7):709-725.e6.

5 Pierce BG, Wiehe K, Hwang H, Kim BH, Vreven T, Weng Z. ZDOCK server: interactive docking prediction of protein-protein complexes and symmetric multimers. Bioinformatics. 2014;30(12):1771-1773.

6 Le Guilloux V, Schmidtke P, Tuffery P. Fpocket: an open source platform for ligand pocket detection. BMC Bioinformatics. 2009;10:168.

7 Trott O, Olson AJ. AutoDock Vina: improving the speed and accuracy of docking with a new scoring function, efficient optimization, and multithreading. J Comput Chem. 2010;31(2):455-461.

8 Hess B, Kutzner C, van der Spoel D, Lindahl E. GROMACS 4: Algorithms for Highly Efficient, Load-Balanced, and Scalable Molecular Simulation. J Chem Theory Comput. 2008;4(3):435-447.

Figure S1

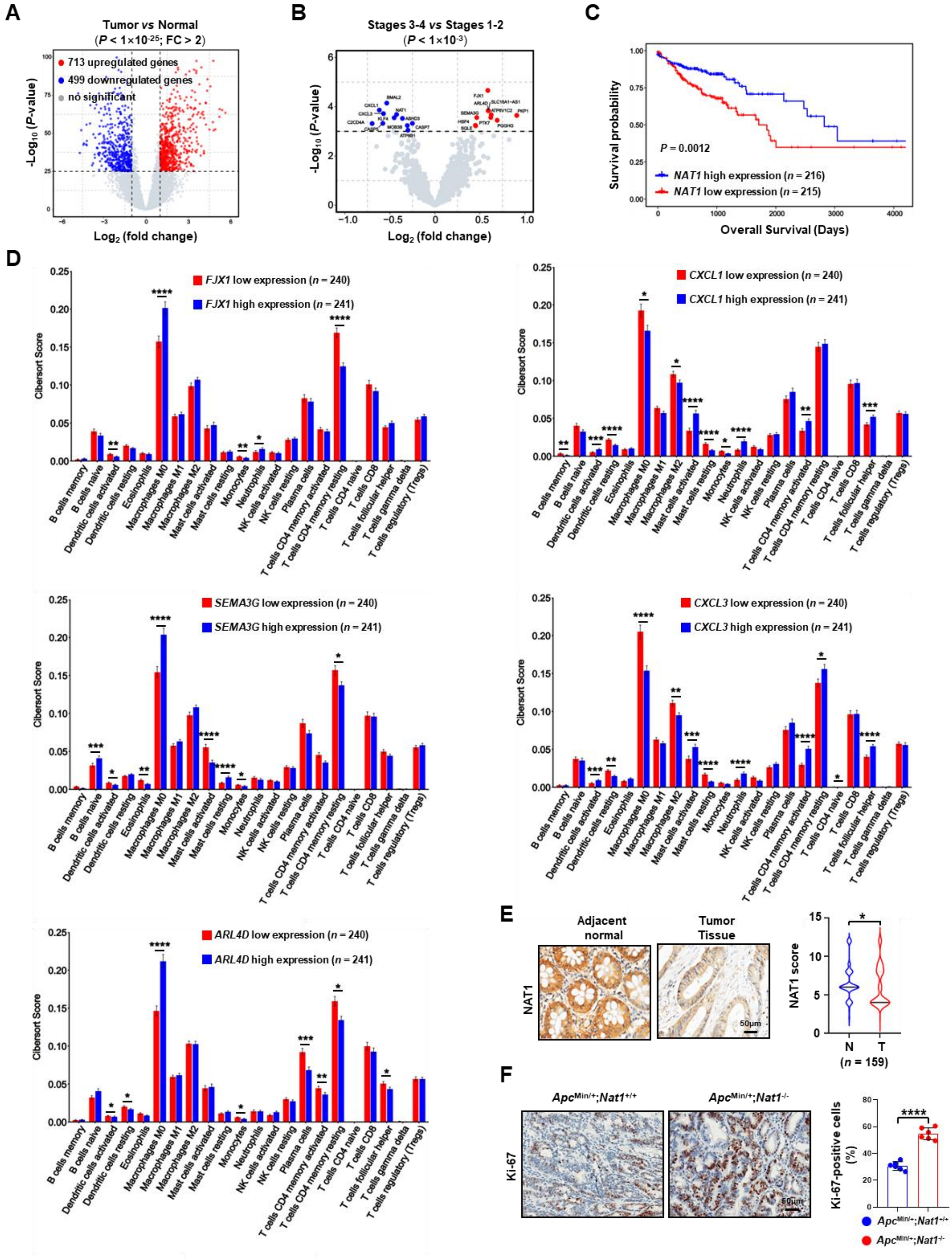

FIGURE S1. Public databases identify NAT1 as a key prognosis-related gene associated with immunity and the progression of colorectal cancer (CRC). (**A**) A volcano plot illustrating the differentially expressed genes (DEGs) identified from the RNA-seq dataset of The Cancer Genome Atlas

Colon Adenocarcinoma (TCGA-COAD), comparing tumor and normal tissues. (**B**) A volcano plot depicting the DEGs in the TCGA-COAD RNA-seq dataset, comparing Stage 3-4 with Stage 1-2. (**C**) A Kaplan–Meier survival curve demonstrates a significant association between low levels of *NAT1* and poor survival in CRC patients (P = 0.0012), with low *NAT1* (n = 215) and high *NAT1* (n = 216) groups. (**D**) The CIBERSORT algorithm was utilized to assess the immune score based on the expression of *FJX1*, *CXCL1*, *SEMA3G*, *CXCL3*, or *ARL4D*. (**E**) Immunohistochemical (IHC) staining for NAT1 in representative CRC tissue samples (left) and violin plots illustrating NAT1 expression in 159 CRC samples (right). (**F**) Representative Ki-67 staining of colon tumors from $Apc^{Min/+}$;$Nat1^{+/+}$ and $Apc^{Min/+}$;$Nat1^{-/-}$ mice (left); the percentage of tumor cells exhibiting Ki-67 positive nuclear immunostaining in the colon tumors from both genotypes (right) (n = 6 per group). Data are presented as mean ± s.d. Survival was analyzed by using the Kaplan–Meier method and compared using the log-rank test (C). P values were determined by a two-tailed Student's t-test (D-F). *P < 0.05, **P < 0.01, ***P < 0.001, ****P < 0.0001.

Figure S2

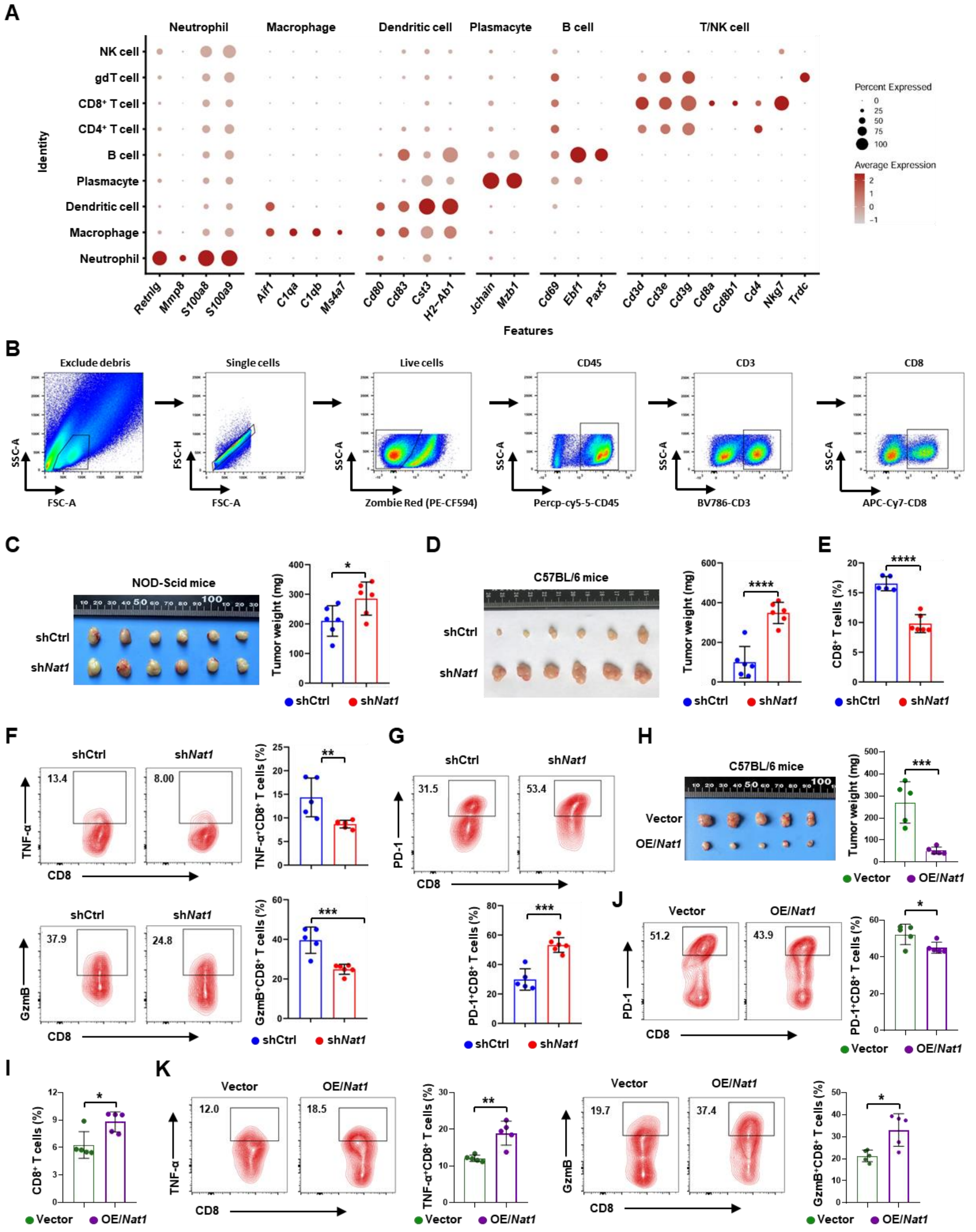

FIGURE S2. NAT1 deficiency impairs the anti-tumor activity of CD8^{+} T cells and promotes tumor growth. (**A**) A bubble plot illustrating selected cell type-specific markers across all identified cell clusters. The dot size represents the proportion of cells expressing each marker. (**B**) Gating strategies and representative flow cytometry plots used to identify the specified immune cell

populations. (**C and D**) Equal numbers of MC38 cells, stably silencing *Nat1* or a control, were subcutaneously injected into either immunodeficient NOD-Scid mice (C) or immunocompetent C57BL/6 mice (D). Representative images of excised tumors (left) and tumor weights (right) are shown (n = 6 per group). (**E-G**) Flow cytometry analysis showing the proportions of $CD8^+$ T cells (E), TNF-α^+CD8^+ T cells and $GzmB^+CD8^+$ T cells (F), as well as PD-1^+CD8^+ T cells (G) in *Nat1*-silenced (n = 6) or control (n = 5) tumors. (**H**) Equal numbers of MC38 cells, stably overexpressing *Nat1* or a control, were subcutaneously injected into immunocompetent C57BL/6 mice. Representative images of excised tumors (left) and tumor weights (right) are shown (n = 5 per group). (**I-K**) Flow cytometry analysis shows the proportions of $CD8^+$ T cells (I), PD-1^+CD8^+ T cells (J), as well as TNF-α^+CD8^+ T cells and $GzmB^+CD8^+$ T cells (K) in *Nat1*-overexpressing (n = 5) or control (n = 5) tumors. Data are presented as mean ± s.d. P values were determined by a two-tailed Student's t-test (C-K). *P < 0.05, **P < 0.01, ***P < 0.001, ****P < 0.0001.

Figure S3

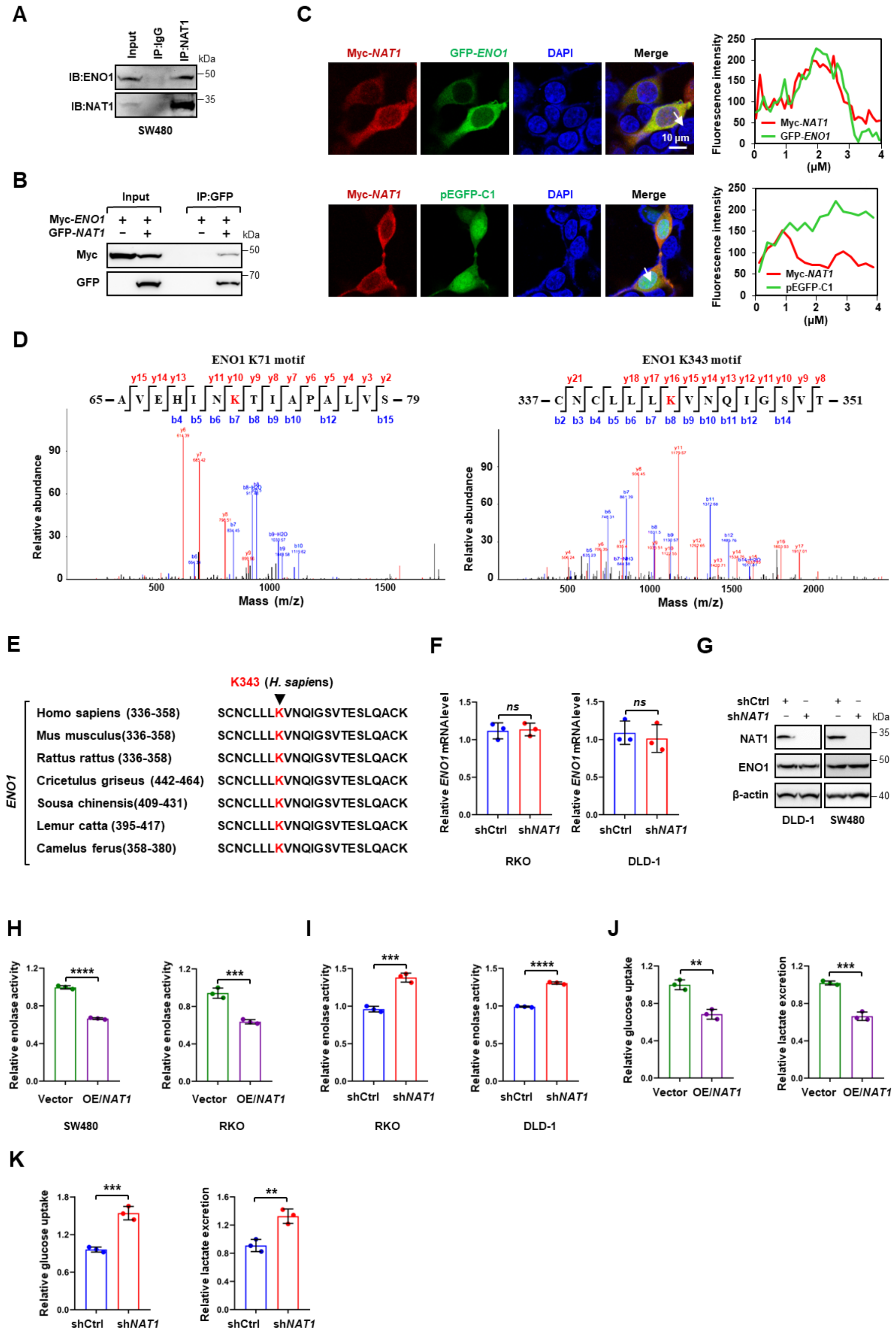

FIGURE S3. NAT1 binds to and acetylates ENO1 which inhibits its enzymatic activity and ultimately reduces glycolysis in CRC. (**A and B**) Co-immunoprecipitation (Co-IP) analyses confirm the endogenous (A) and

exogenous (B) interactions between NAT1 and ENO1. (**C**) Confocal microscopy demonstrates co-localization of NAT1 and ENO1 in SW480 cells. (**D**) Mass spectrometry (MS) identifies the acetylation sites at K71 and K343 of ENO1. (**E**) Alignment of ENO1 amino acid sequences from various species, with residue 343 highlighted in red. (**F**) qPCR analysis shows *ENO1* mRNA levels in *NAT1*-silencing RKO (left) and DLD-1 (right) cells. (**G**) Western blot analysis measures ENO1 protein levels in *NAT1*-silencing DLD-1 (left), and SW480 (right) cells. (**H**) ELISA analysis assesses enolase activity in *NAT1*-overexpressing SW480 (left) and RKO (right) cells. (**I**) ELISA analysis evaluates enolase activity in *NAT1*-silencing RKO (left) and DLD-1 (right) cells. (**J**) ELISA analysis shows glucose uptake (left) and lactate production (right) in *NAT1*-overexpressing RKO cells. (**K**) ELISA analysis shows glucose uptake (left) and lactate production (right) in *NAT1*-silencing DLD-1 cells. Data are presented as mean ± s.d. P values were determined by a two-tailed Student's t-test (F, H-K), $n = 3$ per group. $^{**}P < 0.01$, $^{***}P < 0.001$, $^{****}P < 0.0001$; *ns*, not significant

Figure S4

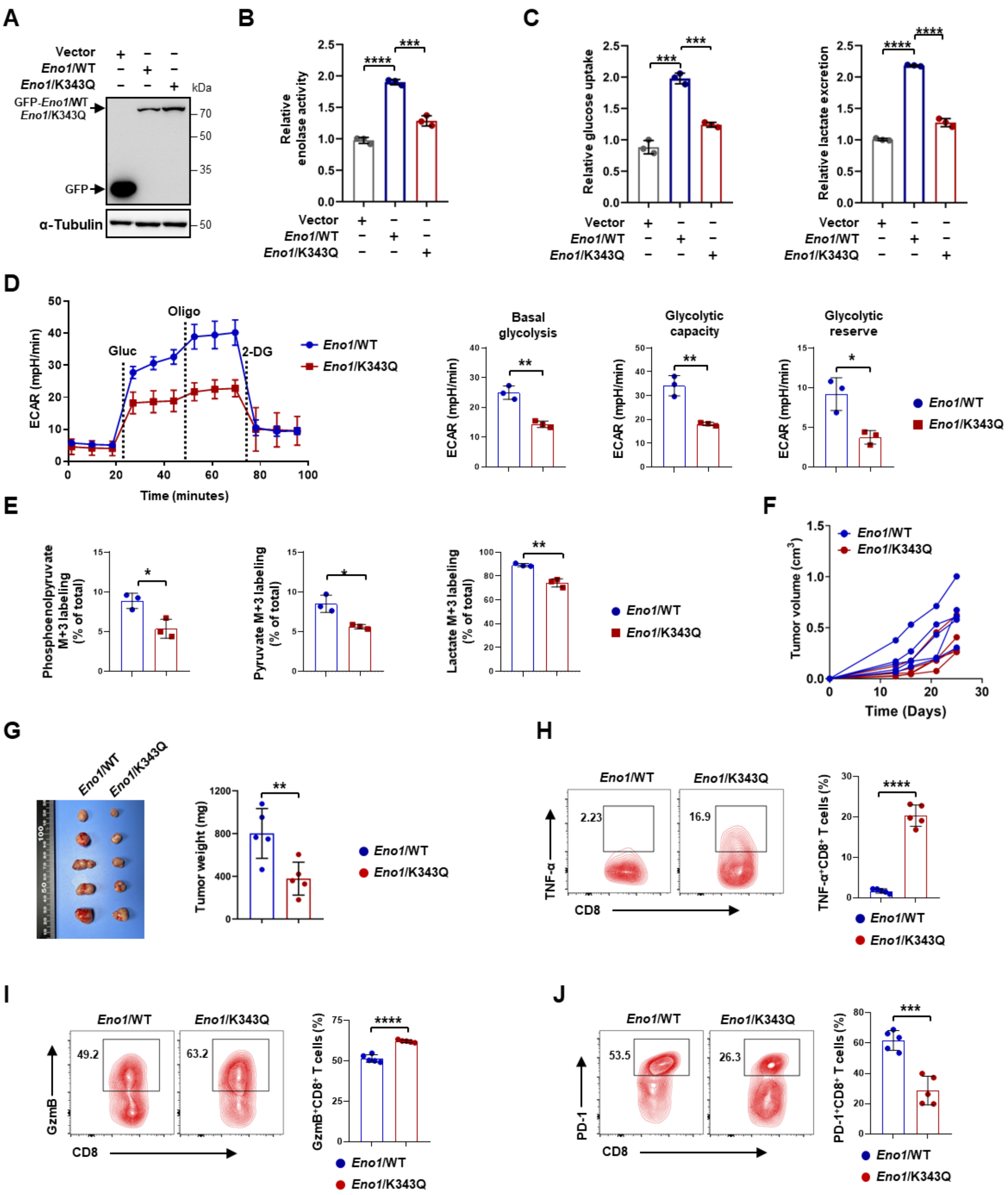

FIGURE S4. Mimicking protein hyperacetylation at ENO1 K343 inhibits its enzymatic activity and lactate production while enhancing T cell anti-tumor immunity. (**A**) Western blot analysis confirms the stable expression of *Eno1*/WT and *Eno1*/K343Q in MC38 cells. (**B and C**) ELISA analysis assesses enolase activity (B) as well as glucose uptake and lactate production (C) in MC38 cells that stably express *Eno1*/WT or *Eno1*/K343Q (*n*

= 3 per group). (**D**) ECAR rates in *Eno1*/WT and *Eno1*/K343Q MC38 cells are shown, with derived parameters including glycolysis, glycolytic capacity, and glycolytic reserve (n = 3 per group). (**E**) Relative isotopomer abundance of phosphoenolpyruvate, pyruvate and lactate in *Eno1*/WT and *Eno1*/K343Q MC38 cells is shown (n = 3 per group). (**F and G**) Equal numbers of MC38 cells stably overexpressing *Eno1*/WT or *Eno1*/K343Q were subcutaneously injected into immunocompetent C57BL/6 mice, and the resulting tumor growth curves (F), representative images of excised tumors (G, left), and tumor weights (G, right) are presented (n = 5 per group). (**H-J**) Flow cytometry analysis shows the proportions of TNF-α^{+}CD8^{+}T cells (H) and GzmB^{+}CD8^{+} T cells (I), as well as PD-1^{+}CD8^{+} T cells (J) in MC38 tumors that stably express *Eno1*/WT or *Eno1*/K343Q (n = 5 per group). Data are presented as mean ± s.d. P values were determined by one-way ANOVA with Tukey's post hoc test (B and C) or a two-tailed Student's t-test (D-J). *$P < 0.05$, **$P < 0.01$, ***$P < 0.001$, ****$P < 0.0001$.

Figure S5

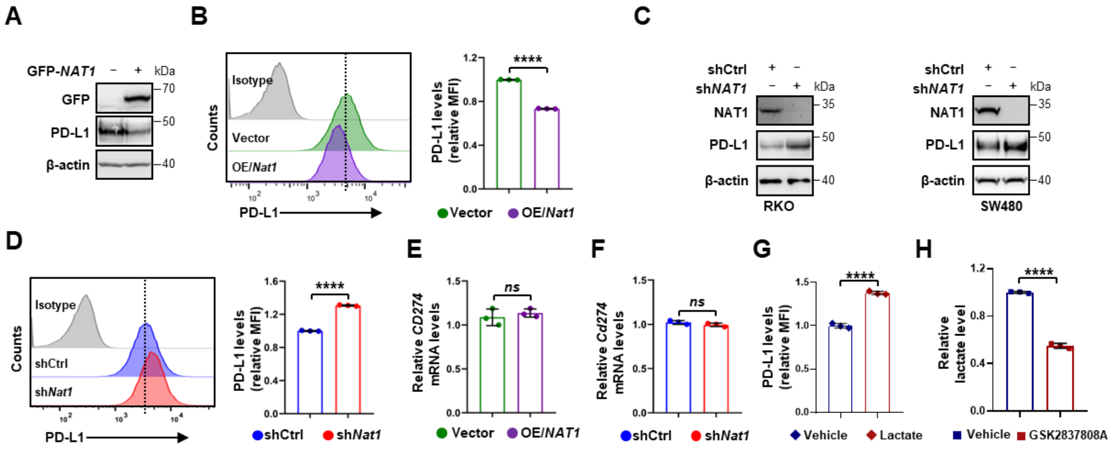

FIGURE S5. NAT1 regulates PD-L1 expression. (**A**) Western blot (WB) analysis demonstrates the levels of PD-L1 expression in RKO cells overexpressing *NAT1*. (**B**) Histograms (left) and summaries of mean fluorescence intensity (MFI) (right) illustrate PD-L1 levels in MC38 cells with *Nat1* overexpression. (**C**) WB analysis reveals the levels of PD-L1 expression in RKO (left) and SW480 (right) cells with *NAT1* silencing. (**D**) Histograms (left) and MFI summaries (right) depict PD-L1 levels in MC38 cells with *Nat1* silencing. (**E and F**) Quantitative PCR (qPCR) analysis indicates *CD274* mRNA levels in RKO cells overexpressing *NAT1* (E) and in MC38 cells with *Nat1* silencing (F). (**G and H**) SW480 cells were treated with lactate (0.5 mM) (G) or GSK2837808A (10 μM) (H) for 15 hours, after which PD-L1 expression was analyzed using flow cytometry. Data are presented as mean ± s.d. *P* values were determined by a two-tailed Student's *t*-test (B, D-H), *n* = 3 per group. ****$P < 0.0001$; *ns*, not significant.

Figure S6

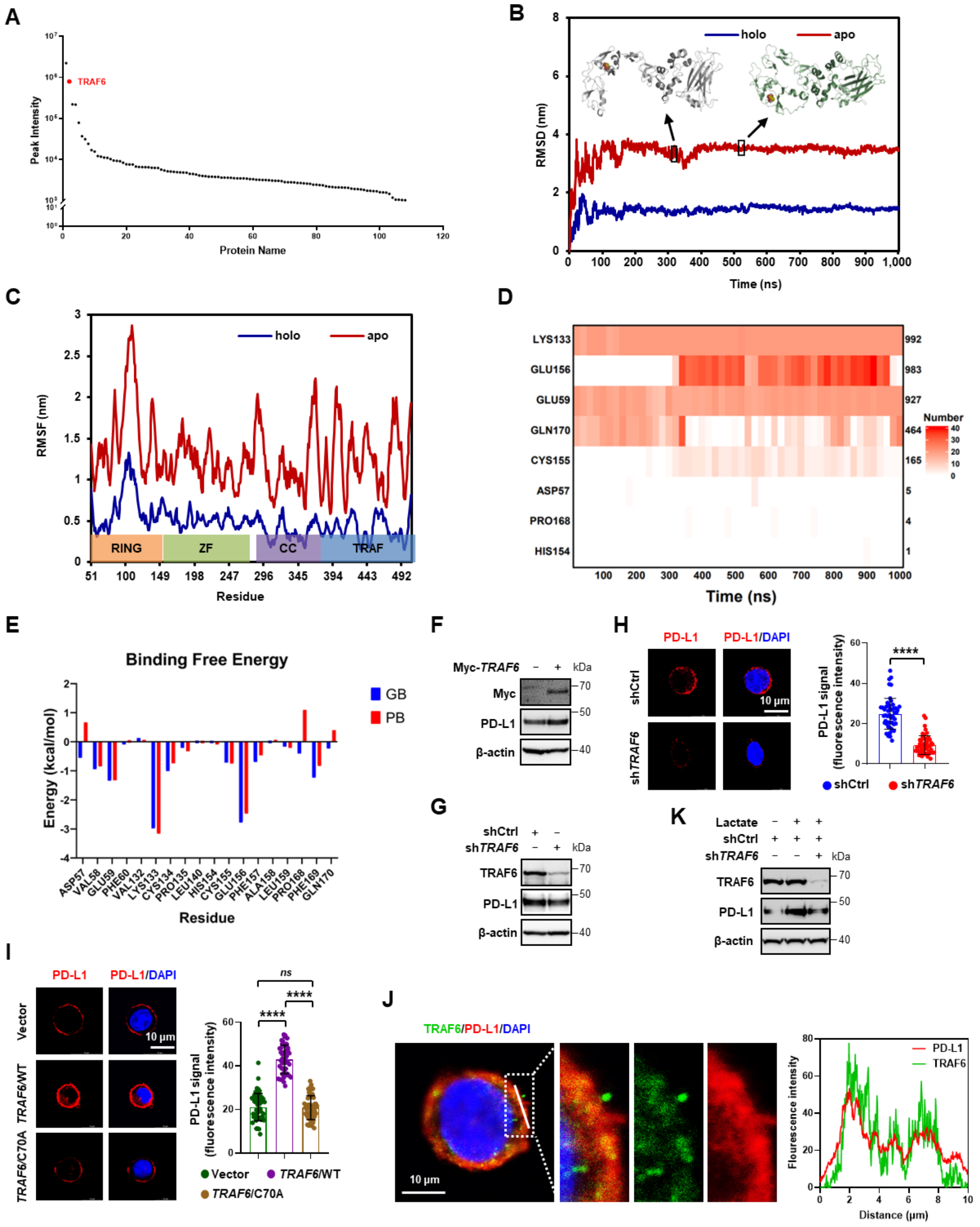

FIGURE S6. Lactate binds to and activates TRAF6 to enhance PD-L1 protein stability. (**A**) Scores of candidate lactate-binding proteins identified through mass spectrometry. (**B**) Root Mean Square Deviations (RMSDs) of the apo and lactate-bound TRAF6 systems. Two representative conformations at 300 ns and 500 ns are depicted in gray and green, respectively. (**C**) Root Mean Square Fluctuations (RMSFs) of the apo and holo (lactate-bound) TRAF6

systems. (**D**) Temporal evolution of the interactions between lactate and TRAF6 residues within a distance of < 3.5 Å. (**E**) Binding free energy contributions of key residues calculated using MM/PB(GB)SA. (**F and G**) Western blot (WB) analysis shows the levels of PD-L1 expression in SW480 cells with *TRAF6* overexpression (F) or silencing (G). (**H and I**) Representative confocal images show PD-L1 expression at the membrane in *TRAF6*-silenced (H), and *TRAF6*/WT-overexpressing or *TRAF6*/C70A-expressing (I) CRC cells. The bar chart shows the quantification of membrane PD-L1 signals (right). (**J**) Confocal microscopy shows TRAF6–PD-L1 colocalization in SW480 cells (left), and the relative fluorescence intensity of TRAF6 and PD-L1 was analyzed (right). (**K**) WB analysis demonstrates PD-L1 protein expression levels in *TRAF6*-silenced or control SW480 cells treated with or without lactate. Data are presented as mean ± s.d. P values were determined using a two-tailed Student's t-test (H) or one-way ANOVA with Tukey's post hoc test (I). ****$P < 0.0001$; *ns*, not significant.
